# Mapping brain function in adults and young children during naturalistic viewing with high‐density diffuse optical tomography

**DOI:** 10.1002/hbm.26684

**Published:** 2024-05-04

**Authors:** Kalyan Tripathy, Morgan Fogarty, Alexandra M. Svoboda, Mariel L. Schroeder, Sean M. Rafferty, Edward J. Richter, Christopher Tracy, Patricia K. Mansfield, Madison Booth, Andrew K. Fishell, Arefeh Sherafati, Zachary E. Markow, Muriah D. Wheelock, Ana María Arbeláez, Bradley L. Schlaggar, Christopher D. Smyser, Adam T. Eggebrecht, Joseph P. Culver

**Affiliations:** ^1^ Division of Biological and Biomedical Sciences Washington University in St. Louis St. Louis Missouri USA; ^2^ Mallinckrodt Institute of Radiology Washington University School of Medicine St. Louis Missouri USA; ^3^ Western Psychiatric Hospital University of Pittsburgh Medical Center Pittsburgh Pennsylvania USA; ^4^ Imaging Science Program Washington University in St. Louis St. Louis Missouri USA; ^5^ Department of Electrical and Systems Engineering Washington University in St. Louis St. Louis Missouri USA; ^6^ Department of Physics Washington University in St. Louis St. Louis Missouri USA; ^7^ Department of Biomedical Engineering Washington University in St. Louis St. Louis Missouri USA; ^8^ Department of Pediatrics Washington University School of Medicine St. Louis Missouri USA; ^9^ Kennedy Krieger Institute Baltimore Maryland USA; ^10^ Department of Neurology Johns Hopkins University School of Medicine Baltimore Maryland USA; ^11^ Department of Pediatrics Johns Hopkins University School of Medicine Baltimore Maryland USA; ^12^ Department of Neurology Washington University School of Medicine St. Louis Missouri USA

**Keywords:** brain development, feature regressor analysis, functional near‐infrared spectroscopy, movie viewing, optical neuroimaging

## Abstract

Human studies of early brain development have been limited by extant neuroimaging methods. MRI scanners present logistical challenges for imaging young children, while alternative modalities like functional near‐infrared spectroscopy have traditionally been limited by image quality due to sparse sampling. In addition, conventional tasks for brain mapping elicit low task engagement, high head motion, and considerable participant attrition in pediatric populations. As a result, typical and atypical developmental trajectories of processes such as language acquisition remain understudied during sensitive periods over the first years of life. We evaluate high‐density diffuse optical tomography (HD‐DOT) imaging combined with movie stimuli for high resolution optical neuroimaging in awake children ranging from 1 to 7 years of age. We built an HD‐DOT system with design features geared towards enhancing both image quality and child comfort. Furthermore, we characterized a library of animated movie clips as a stimulus set for brain mapping and we optimized associated data analysis pipelines. Together, these tools could map cortical responses to movies and contained features such as speech in both adults and awake young children. This study lays the groundwork for future research to investigate response variability in larger pediatric samples and atypical trajectories of early brain development in clinical populations.

## Graphical abstract

High‐density diffuse optical tomography (HD‐DOT) is an emerging neuroimaging approach combining logistical advantages of optical imaging with image quality benchmarked against fMRI. We developed a child‐friendly high‐performance HD‐DOT system, a children's movie stimulus library, and associated data analysis pipelines. Integrating these tools, we mapped speech responses in awake young children.

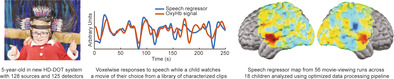

**Abbreviations**
fMRI: functional magnetic resonance imaging
fNIRS: functional near‐infrared spectroscopy
GVTD: global variance of temporal derivatives
HD‐DOT: high‐density diffuse optical tomography

## 1 INTRODUCTION

The first several years of human life are packed with exciting behavioral changes, but the underlying changes in brain function have been challenging to study. Since the pioneering work of Hubel and Wiesel using the cat visual system as a model, decades’ worth of animal experiments have provided valuable insight into the sensitive periods for brain development and heightened neuroplasticity that occur early in life (Rahn et al., 2022; Reh et al., 2020; Wiesel & Hubel, 1963, 1965). However, not all human behaviors are readily investigated in animal models. For instance, human language, with its complex structure and compositional nature, is distinct from other forms of animal communication (Pagel, 2017). Furthermore, while laboratory models provide opportunities for powerful basic science research, translation into clinical neuroscience studies in patient populations requires methods for measuring brain function in humans.

Human studies of functional brain development have been constrained by available imaging methods and their tradeoffs between image quality and logistics. Functional magnetic resonance imaging (fMRI) has been used to map brain function in adults in exquisite detail, but the confined, loud, and solitary scanning environment and low tolerance for head motion are not conducive to scanning young children while they are awake (Raschle et al., 2012). As a result, there have been very few studies of the development of language or other behaviors in children younger than 4 years (Vanderwal et al., 2019; Weiss‐Croft & Baldeweg, 2015; Yu et al., 2021). Alternative recording methods circumvent some of these limitations of fMRI at the expense of their own shortcomings. For instance, electroencephalography (EEG) can be used to record electrical signals from the cortical surface in diverse populations and settings, but is particularly limited by its spatial resolution. Meanwhile, optically pumped magnetometer magnetoencephalography (OPM‐MEG) has been applied in fascinating studies of pediatric populations and naturalistic tasks as a new, wearable alternative to traditional MEG or fMRI, although data collection requires specially designed imaging suites with expensive magnetic shielding (Boto et al., 2022; Brookes et al., 2022; de Lange et al., 2021; Feys et al., 2022; Hill et al., 2019). Functional near‐infrared spectroscopy (fNIRS) is another alternative imaging modality that uses light to track brain function in an open, silent, child‐friendly environment. Several studies have used the current functional neuroimaging gold standard of fMRI to validate fNIRS as an effective means of localizing and tracking hemodynamic signals (Cui et al., 2011; Huppert et al., 2006; Wijeakumar et al., 2017). The potential of fNIRS in developmental neuroscience research is illustrated by studies of the early development of language (Butler et al., 2020), face recognition (Otsuka et al., 2007) and social processing (Hakuno et al., 2018), among other behaviors (Pinti et al., 2020). These studies have shown lateralized differences in brain activity between various task states in typically and atypically developing children (Butler et al., 2020; Otsuka et al., 2007). However, the traditionally sparse sampling of fNIRS limits its image quality, diminishing the resolution and accuracy with which differences between ages, groups, and task conditions are localized in the brain (Fishell et al., 2020; White & Culver, 2010).

High‐density diffuse optical tomography (HD‐DOT) is an emerging neuroimaging approach that uses dense arrays of light sources and detectors to combine logistical advantages of optical imaging with tomographic reconstruction, increased resolution, reduced localization artifacts, and other image quality improvements relative to traditional fNIRS (Fishell et al., 2020; Frijia et al., 2021; White & Culver, 2010; Zeff et al., 2007). HD‐DOT has been extensively benchmarked against fMRI (Eggebrecht et al., 2012, 2014) and has potential to be a powerful tool for developmental neuroscience research. However, prior HD‐DOT studies have focused on adults (Eggebrecht et al., 2014), children older than 7 years (Fishell et al., 2020), or infants (Ferradal et al., 2016; Frijia et al., 2021; Liao et al., 2012), omitting the intermediate age range of 1–7 years. Imaging these young children while awake requires optimization of instrumentation for this age group as well as accompanying child‐friendly task paradigms. Movies can serve as rich stimuli for mapping brain function (Bartels & Zeki, 2004; Fishell et al., 2019; Hasson et al., 2004) while improving compliance and reducing head motion in children (Greene et al., 2018; Vanderwal et al., 2019). Here, we further developed optical imaging methods previously applied in adults, characterized movie stimuli and data analysis methods initially employed by fMRI studies, and combined these tools to map responses to naturalistic speech in awake young children.

We first developed an HD‐DOT system targeted towards imaging preschool‐age children with an increased channel count and expanded field of view among other image quality improvements over previous systems. System performance was validated based on raw data quality and reconstructed image quality for conventional task data from a group of adults. Next, we compiled and characterized a library of children's movie clips and associated data analysis pipelines, developing and validating these tools in a pool of highly sampled adults. Finally, the optimized system, stimulus library, and analysis pipelines were used to map receptive language function during movie viewing in a pilot group of children. We therein establish the feasibility of mapping brain function in young children while awake with HD‐DOT. Our work lays a critical methodological foundation for future research to further investigate typical and atypical trajectories of functional brain development through larger cross‐sectional and longitudinal studies in various pediatric populations.

## 2 METHODS

### 2.1 Preschooler HD‐DOT system design and build

The study aimed to first develop an optical neuroimaging system geared towards imaging children while maximizing data quality. To achieve this goal, the system was designed and built as follows:

#### 2.1.1 Illumination

The instrument illuminated the head with 128 laser sources at each of two wavelengths, 685 and 830 nm (HL8338MG and HL6750MG, Thor Labs). Light levels were recorded for each source at 685 nm and averaged 0.39 ± 0.04 mW/mm^2^, well within American National Standards Institute consensus standards for near‐infrared light exposure.

#### 2.1.2 Detection

Scattered light was measured by 125 avalanche photodiode (APD) detectors (C12703‐01 SPL, Hamamatsu), and each one's signal was digitized by a dedicated analog‐to‐digital converter (ADC) at 96 kHz (A16R, Focusrite).

#### 2.1.3 Control of illumination and detection

Light sources were controlled by a dedicated source computer, using software created in‐house and connected to a 20 MHz digital input/output card (782608–01, National Instruments) and two BrainBoxes for multiplexing and demultiplexing of signals, as detailed for previous systems (Eggebrecht et al., 2014). This setup enabled sources to be illuminated in highly temporally precise encoding patterns, allowing time and frequency coding of the source responsible for every detected signal. Detected and digitized signals were sent from the ADCs to a separate detector computer, on which another custom‐coded software program presented real‐time displays of light and noise levels and allowed for initiation and termination of data collection. For each time encoded step, a period of 104 μs between measurements allowed for detectors to recover from saturation and the analog‐to‐digital converter to settle, such that signals adjusted to the new light level without measurements bleeding in between time points.

#### 2.1.4 Imaging fibers

**FIGURE 1:**
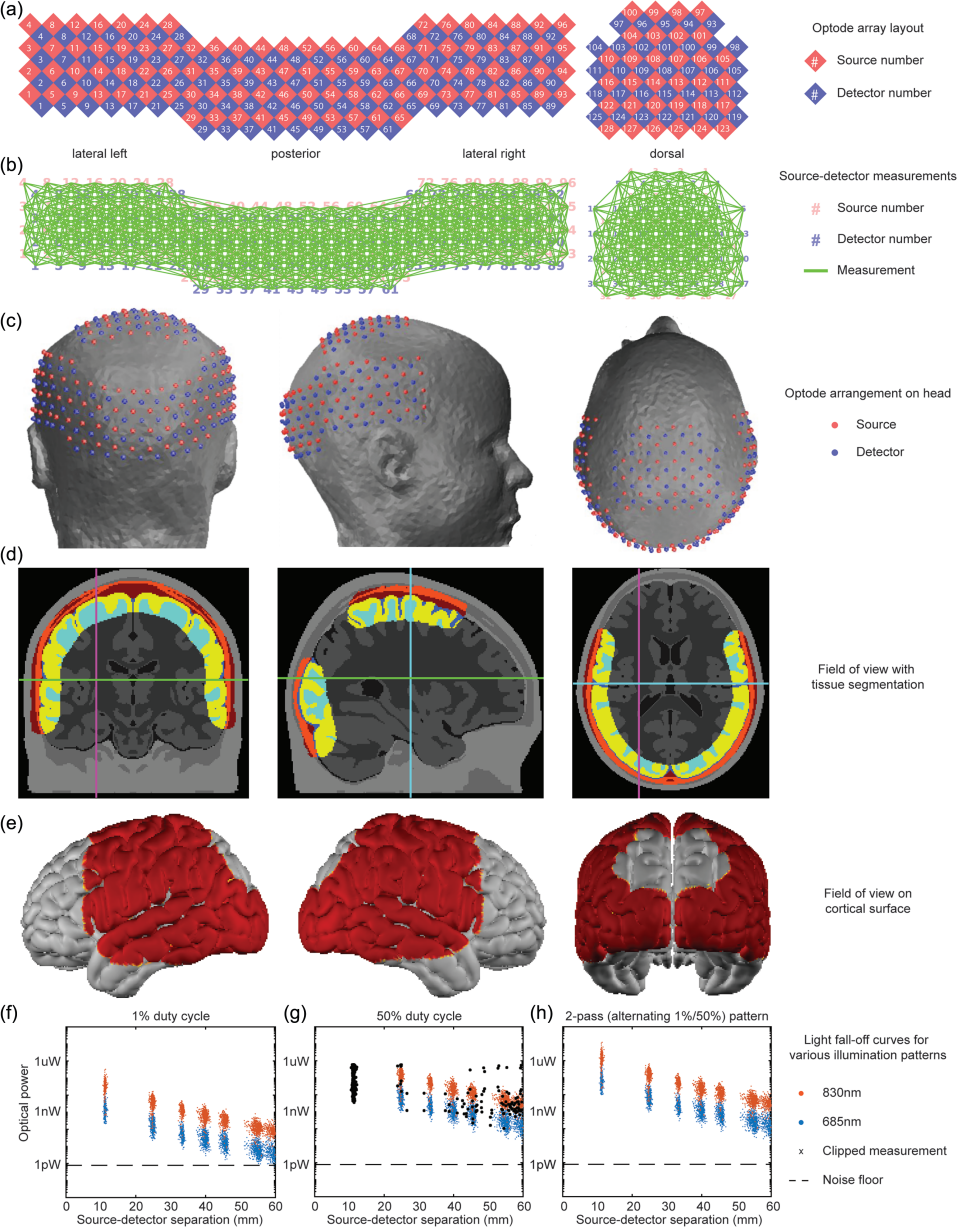
Preschooler HD‐DOT system: (a) A schematic of the optode array with 128 sources (red) and 125 detectors (blue). (b) A depiction of the high‐density measurements – each green line represents one of 2464 source‐detector pairs with <50 mm separation. (c) Source and detector positions on the head during a typical imaging session. (d) Tomographic slices of a flat field reconstruction thresholded at 10% of its maximum illustrate the depth of sensitivity. (e) Surface projection of a flat field reconstruction illustrates the cortical coverage typically achieved with the imaging cap, including parts of frontal, parietal, temporal, and occipital cortex. (f–h) Light fall‐off curves for data collected on an optical head phantom with various source encoding patterns. (f) Illumination with a 1% duty cycle allows for unsaturated nearest‐neighbor measurements but low light levels approaching the noise floor for long‐range measurements. (g) With a 50% duty cycle, light levels remain higher above the noise floor at longer distances but are frequently clipped at shorter distances. (h) A 2‐pass encoding pattern alternating between 1% and 50% duty cycles supports unclipped measurements for short‐range separations and measurements well above the noise floor for longer separations, maximizing dynamic range.

Source, APD, and ADC boxes were stacked on a mobile extruded aluminum cart (custom design, MiniTec; Supplemental Figure S1A–B). Light was conveyed between the participant's head and the sources and detectors themselves by bundles of fiber‐optic cables (FTIIQ24425 for connecting two sources, one for each wavelength, to each source position on the cap, and FTIIG24426 for 1:1 detector connections, both from Fiberoptics Technology). The weight of the fibers was supported by and symmetrically distributed around a pair of concentric, suspended wooden halos (12 “x 5/4” wood disks with an 8″ cutout, Menards) and a mobile, extruded aluminum frame (custom design, MiniTec; Supplemental Figure S1C). Fiber tips were capped with 3‐D printed, round‐ended, soft plastic sleeves secured by heat‐shrink tubing to reduce the sharpness of optodes on the scalp (Supplemental Figure S1D). Fiber ends were held in place by an imaging cap in a grid of alternating source and detector positions that curved around the head surface (Figure 1a–c).

#### 2.1.5 Imaging cap

A model head was created by 3‐D printing a structural MR image from a 1 year‐old child (Fonov et al., 2009, 2011), as this was the youngest age we aimed to image. An adjustable, multi‐panel cap design and flexible materials were chosen so the cap would fit a range of head sizes and shapes. The main body of the cap was made by molding four heated panels of thermoplastic Aquaplast (NC81339‐1, North Coast Medical) to four surfaces (occipital, left temporal, right temporal, and dorsal) of the model head. The Aquaplast panels were allowed to cool and harden before lining their inner surfaces with soft foam. The occipital panel was attached to each of the two temporal panels by a series of zip ties, while the dorsal panel was kept separate. The chosen thermoplastic material and this multi‐panel design ensured a combination of structural integrity across long‐term usage and flexibility to fit the cap to multiple head shapes and sizes. A uniform grid of holes with 11 mm spacing was drilled across the formed panels, creating first‐through fifth‐ nearest source‐detector separations of 1.1, 2.5, 3.3, 3.9 and 4.6 cm. The holes were then lined with 3‐D printed, cylindrical plastic guides, secured by rubber bands, to hold fibers perpendicular to the head surface. The cap panels were suspended by string from the wooden halos, and then fibers were inserted in their designated positions through the guides. Fibers were held in place by elastic foam rings (punched out of a sheet, 8785 K82, McMaster Carr) that also provided a spring action to press fiber tips against the participant's scalp. Fiber ends projected 5 mm beyond the inner surface of the cap so that they could be combed through hair like the teeth of a hairbrush (Supplemental Figure S1E). Velcro straps and buckles were added along the edges of the cap so that the panels could be fastened in fixed positions relative to each other and pressed close against the head during imaging sessions. Designing the cap with four distinct panels connected by Velcro straps improved scalp‐to‐optode coupling relative to a rigid single‐piece design, since the separated cap panels – with some inherent flexibility offered by their thermoplastic composition and adjustable relative positioning offered by the straps connecting them – could better contour to varying shapes and curvatures of the head surface.

#### 2.1.6 Ancillary equipment and imaging suite

The cap was suspended above a motorized salon chair that could be lowered or raised according to the participant's height. A child‐sized salon chair was used for children with a five‐point harness to secure younger children while an adult‐sized salon chair was used for scanning adults. The height of the cap could also be changed by adjusting the frame but was generally held fixed across imaging sessions, except for the dorsal panel that was lowered and raised each time a participant entered and left the cap. Residual slack in the fibers allowed participants to make small postural adjustments during breaks in scanning sessions. An LCD display was positioned at eye level 90 cm away from the cap to present visual stimuli and two speakers placed at the opposite end of the room were used to deliver auditory stimuli with stereo sound. A screen‐based eye tracker (X3‐120, Tobii Pro) was mounted below the display for a subset of imaging sessions. Seven cameras were mounted around the aluminum frame to capture the position of the cap on the participant's head during each imaging session from upper and lower viewing angles on the left, right, and back of the cap as well as head‐on. The aluminum frame was cushioned and decorated with colored foam to have the appearance of a sandcastle, the aluminum cart was covered with blue curtains, and the walls and doors of the room were covered with stickers to give the entire imaging suite an under‐the‐sea theme (Supplemental Figure S1A). There was also ample space for one or more additional chairs to accommodate caregivers and experimenters sitting near children for comfort and supervision during imaging sessions.

#### 2.1.7 Stimulus delivery

A third computer used the Psychophysics Toolbox 3 package for MATLAB (Brainard, 1997) to present stimuli to participants via an audiovisual mixer connected to the two speakers and display. Synchronizing signals were sent from the source computer via the demultiplexing boxes and from the stimulus computer via the audiovisual mixer to the detector computer for time‐locked alignment of source, detector, and stimulus information.

### 2.2 Cap fit procedure

The main goals of the cap fit process were to get the large number of optodes through the hair and in direct contact with the scalp surface, position the cap consistently across sessions, and maximize participant comfort. These objectives were fulfilled through a systematic cap fit process, featuring modifications to previously described protocols (Eggebrecht et al., 2014; Tripathy et al., 2021) in order to address the expanded field of view (Supplemental Figure S2). Every step of the procedure emphasized hair management, cap placement with reference to fiducial markers, and communication with the participant to enlist their help and ensure their comfort. At the beginning of each cap fit, any participant with long hair had their hair parted down the middle and back and then tied in left and right pigtails (Supplemental Figure S2A,B). The participant was then asked to sit in the imaging chair and slide their head back into the cap, and the pigtails (if present) were threaded between the lateral and dorsal cap panels to minimize obstruction of light transmission between optodes and the scalp (Supplemental Figure S2C). The height of the chair was adjusted to position the participant's ears just below the lateral panels of the cap. The participant was then directed to hold a pair of Velcro straps at the front of the cap and move the cap and their head side‐to‐side to comb the optodes through their hair and against the surface of the scalp (Supplemental Figure S2D). The tragus on either side of the head and fiducial markers on the cap were used as points of reference to ensure the cap was positioned symmetrically and consistently, and the front Velcro straps were then fastened (Supplemental Figure S2E,F). The dorsal imaging panel was lowered by an experimenter, combed through the hair on the top of the head, and then centered and aligned with the rest of the cap by referencing fiducial markers on both the cap (e.g., color‐coded rings around designated optodes) and the participant (e.g., the nasion). Velcro straps were used to secure the dorsal panel relative to the rest of the cap (Supplemental Figure S2G,H). Real‐time data quality visualizations and tactile feedback from the participant were then utilized to guide any further combing of individual occluded optodes with low light levels through obstructing hair. The final position of the cap on the participant's head was recorded from seven camera angles.

For child imaging sessions, additional time was allotted at the outset for participants to become acquainted with the experimenters and the imaging environment. Once children appeared sufficiently comfortable, an animated movie clip was played to keep them engaged, and the help of caregivers was enlisted as needed for the cap fit procedure. Experimenters lightly combed optodes through participants’ hair and aligned the cap using the ears, tragus, and fiducials on the cap as markers of height and symmetry. Real‐time data quality visualizations were briefly evaluated before beginning data collection. In total, the time taken for set up from participant arrival to starting data collection targeted 15 min for adult participants and 30 min for child imaging sessions, including habituation to the environment, hairdo, and cap fit.

### 2.3 Participants

We first validated the performance of our new imaging system, task paradigm, and analysis pipeline in young, healthy adults as a cooperative, high‐performing, well‐characterized reference population. In the first phase of the study (HD‐DOT system validation), we mapped responses to visual stimuli, auditory stimuli, and a motor task in a cohort of 5 adults (age range 20–33 years; Supplemental Table 1). In the second phase (movie paradigm validation), we mapped responses to a library of animated movies in three highly sampled adults (Supplemental Table 1). In phase three (child imaging), we attempted to image 31 children (age range 18–81 mo) while they were presented with movies and spoken word lists. Among them, 30 children complied with the cap fit procedure and wore the cap for at least 5 min of imaging. Our inclusion criteria for further analysis were that participants provide at least one high quality movie viewing or word‐hearing task run, where data quality was evaluated based on light levels, measurement variance and retention, pulse signal‐to‐noise ratio, and indicators of head motion (as later detailed in Section 2.6). Altogether, 23 children (age range 23–81 mo) met our criteria for inclusion in final analyses (Supplemental Table 2).

Informed consent was obtained from all adult participants. For children, informed consent was obtained from the participant's parent or legal guardian, and assent was obtained from the child. Consent procedures were conducted in accordance with the IRB protocol approved by the Human Research Protection Office at Washington University School of Medicine.

### 2.4 Stimulus protocols

#### 2.4.1 Visual stimulation (checkerboard viewing)

Participants were asked to sit still and maintain visual fixation on a crosshair at the center of the display while a wedge‐shaped flickering checkerboard stimulus rotated around the screen (Supplemental Figure S3). The black‐and‐white checkerboard pattern reversed at 8 Hz against a constant 50% grey background. The wedge subtended a polar angle of 60°, a radial angle from 2.5° to 10°, and rotated 10° at a time through 36 positions spanning 360° at 1 s per position, for a total of 10 cycles per run (Tripathy et al., 2021).

#### 2.4.2 Auditory stimulation (word hearing)

Participants were asked to sit still and maintain visual fixation on a centrally presented target throughout the task. A simple fixation cross was presented to adults, while children were presented with a cartoon gif to hold their attention. Meanwhile, spoken word lists were presented through the speakers at 1 word per second for 15 s per block, with 15 s of silence in between blocks, and a total of 6 blocks per run. Participants were instructed to passively listen to the spoken words (Eggebrecht et al., 2014).

#### 2.4.3 Motor task (finger tapping)

Participants were asked to sit still and maintain visual fixation on a centrally presented crosshair until either the letter “L” or the letter “R” was presented on the screen. In response to the letter “L”, participants were instructed to tap the fingers of their left hand against their left thumb at 2 Hz. In response to the letter “R”, participants were instructed to perform the same motion with their right hand. The letters were presented for 10 s at a time in a pseudorandomized order, interspersed with rest periods of 24 s, for a total of eight finger tapping blocks per side per run.

#### 2.4.4 Movie viewing

After an initial fixation period of 15 s, during which a silent gif from the movie Finding Nemo was presented on the screen, participants were presented with a 5–6 min‐long audiovisual movie clip. The presented clip was selected from a set of 20 options taken from five different children's movies and TV shows, intended to cover a range of ages and child preferences: Moana, Frozen, Finding Nemo, Curious George, and Daniel Tiger's Neighborhood. Adult participants were presented with all movie clips over the course of multiple imaging sessions: generally, four different clips were presented twice at each of five sessions. Children were allowed to select a movie/show from our library, and were presented with 2–4 alternating clips from that program until they requested that the session end or up to a maximum of 8 movie‐viewing runs interspersed with 2 word‐hearing task runs per imaging session.

### 2.5 Structural MRI data acquisition

Structural MRI data were collected from adult participants to construct subject‐specific head models. MRI scans were conducted on a Siemens Magnetom PRISMA Fit 3.0 T scanner, with an iPAT compatible 20‐channel head coil. Anatomical T1‐weighted MPRAGE scans were collected with echo time = 3.13 ms, repetition time = 2400 ms, flip angle = 8°, and 1 × 1 × 1 mm isotropic voxels. T2‐weighted scans were collected with echo time = 84 ms, flip angle = 120°, and 1 × 1× 4 mm voxels.

### 2.6 HD‐DOT data processing

#### 2.6.1 Pre‐processing

Raw light levels were converted to log ratios across time relative to the temporal mean of each measurement as a baseline. Channels with >7.5% temporal standard deviation across a run were omitted from further analysis to minimize contamination of more subtle hemodynamic signals by non‐physiological nuisance signals such as head motion (Eggebrecht et al., 2014). Data were high‐pass filtered using a 0.02‐Hz cutoff to reduce long‐term drift. Superficial signal regression was performed by averaging all first‐nearest‐neighbor measurements, which sample mostly the scalp, to estimate global systemic signals, and then linearly regressing this out of every source‐detector measurement time trace (Gregg et al., 2010; Zeff et al., 2007). Low‐pass filtering was done with a 0.5‐Hz cutoff to remove residual pulse and other high‐frequency noise. Data were finally downsampled to 1 Hz (Eggebrecht et al., 2014).

#### 2.6.2 Light modeling and reconstruction

Pre‐processed data were reconstructed using an anatomical light propagation model. Generic atlas‐based head models have been shown to provide a viable alternative to optimized subject‐specific head models when subject‐specific anatomical data cannot be acquired (Ferradal et al., 2014). Therefore, pilot data analysis in children used a generic head model made from one adult participant's structural MRI data for simplicity of initial analysis. This head model was based off the adult participant with the smallest head circumference and provided a valuable first approximation for straightforward processing and reconstruction of pediatric neuroimaging data. Future studies will aim to further refine and compare reconstructions using a complete set of child‐specific and/or age‐specific head models spanning the full range of ages imaged. However, the scale of that undertaking was considered beyond the scope of the current project focused on development and validation of the imaging system and paradigm. For the analysis of adult data, wherein each participant's structural MRI data was available, subject‐specific head models were generated for optimized reconstructions. Head models were created using FreeSurfer to segment a T1 image (Dale et al., 1999; Fischl et al., 2001; Ségonne et al., 2004), NIRVIEW to generate a mesh of the participant's head, finite element modeling with spring relaxation approaches to position sources and detectors, and the NIRFAST toolbox to model the diffusion of photons through the head (Dehghani et al., 2003, 2008; Eggebrecht et al., 2014). For subject‐specific head models, photographs from each individual's imaging sessions were used to guide initial placement of the optode array on the head mesh (Supplemental Figure S4A). While head modeling begins with photometric measures, the use of functional localizers has been shown to further improve alignment of optode positions to the head structure. Reconstructions of auditory task data using the first iteration of each head model were thus compared to a reference group fMRI task activation map (Sherafati et al., 2020) to iteratively guide adjustment of head models using Dice coefficient as a measure of overlap (Supplemental Figure S4B). The cap was repositioned and rotated to adjust optode placement on the head mesh, guided by both session photographs and auditory localizer maps, until the Dice coefficient between the thresholded (25% maximum) and binarized HD‐DOT auditory task activation map and reference group fMRI map was greater than 0.10 across the field of view. To avoid reusing localizer data for both head modeling and subsequent evaluation of imaging performance, we restricted the localizers used for cap placement to only a subset of auditory task runs. This approach allowed the remaining auditory, motor, visual, and movie viewing tasks to stay independent from this cap placement procedure and reduce bias when evaluating image reconstructions.

Each resulting sensitivity matrix A is a linear transformation between x, the vector of absorption coefficients within the brain volume for both wavelengths, and y, the vector of relative changes in light levels measured at the head surface, for each time point, in accordance with the linear Rytov approximation:

(1) y=Ax

Sensitivity matrices were inverted as detailed in prior literature (Eggebrecht et al., 2014), here using a Tikhonov regularization parameter *λ*_1_ = 0.05 and a spatially variant regularization parameter *λ*_2_ = 0.1. Traditional Tikhonov regularization penalizes contrast in deeper tissues. To mitigate this issue and reduce associated localization errors during reconstruction, we employed spatially variant regularization (Culver et al., 2003). Detailed image reconstruction methods were previously developed through comparisons between HD‐DOT and fMRI data in healthy adults (Eggebrecht et al., 2014). The current reconstruction parameters were set following those methods, with regularization parameters optimizing the signal‐to‐noise ratio of flat field reconstructions across source‐detector pairs <50 mm separation for the current system. The resultant flat‐field reconstructions were used to designate voxels with sufficient sensitivity (Dehghani et al., 2009), and FreeSurfer segmentation results were used to mask out signals from superficial tissues (i.e., by setting non‐grey matter signals to zero). The intersection across three highly sampled participants of flat‐field reconstructions thresholded at 1% of their maxima was used to designate a common field of view for visualization purposes, as in previous studies (Hassanpour et al., 2015). For quantitative comparisons of different maps, a more stringent sensitivity cutoff was applied for each head model by thresholding its flat‐field reconstruction at 10% of its maximum value to generate a conservative, subject‐specific spatial mask.

#### 2.6.3 Spectroscopy

Relative changes in oxy‐ and deoxy‐ hemoglobin concentrations were calculated from the differential absorption image x at each time point through spectral decomposition:

(2) $$∆C=E^{-1}x$$

where ∆C is a vector of oxy‐ and deoxy‐ hemoglobin concentration changes across the brain volume, and *E* is a matrix of extinction coefficients of oxy‐ and deoxy‐ hemoglobin (Eggebrecht et al., 2014; Tripathy et al., 2021).

#### 2.6.4 Motion censoring

Following best practices outlined by the fMRI literature, we performed censoring of high motion frames after reconstruction (in addition to the superficial signal regression performed during pre‐processing) to prevent corruption of neuroimaging data analysis by motion artifact (Ciric et al., 2017; Power et al., 2012; Siegel et al., 2014). For every run of data, the global variance of temporal derivatives (GVTD) metric of head motion was calculated at each time point as the root‐mean‐square of the temporal derivatives of all measurements (Sherafati et al., 2020):

(3) $$g_{t}=\sqrt{\frac{1}{N}\sum_{m=1}^{N}{\left(y_{\mathrm{mt}}-y_{m\left(t-1\right)}\right)}^{2}}$$

where $g_{t}$ is the GVTD value at time t, m indexes measurements, N is the total number of measurements, and $y_{\mathrm{mt}}$ is the optical density change for measurement m at time t. Time points for which $g_{t}$ rose above a statistically determined threshold $g_{\text{thresh}}$ were censored from further analysis. The value of $g_{\text{thresh}}$ varied between participants, sessions, and runs due to differences in the baseline resulting from physiological variation, but was set through a data‐driven method (Sherafati et al., 2020):

(4) $$g_{\text{thresh}}=\kappa +10\sigma_{L}$$

where κ is the mode of GVTD values and $\sigma_{L}$ is the standard deviation calculated across the number $n_{L}$ of GVTD values less than κ as:

(5) $$\sigma_{L}=\sqrt{\frac{1}{n_{L}}\sum_{g_{t}<\kappa }{\left(g_{t}-\kappa \right)}^{2}}$$

#### 2.6.5 Data quality evaluation

The quality of every data set was evaluated with regard to its light fall‐off curve, the proportion of measurements retained below the 7.5% temporal standard deviation cutoff, GVTD time course, pulse signal‐to‐noise ratio (SNR) across the cap (computed as the ratio of signal power in the 0.5–2 Hz frequency band to the bandwidth‐scaled median power in flanking frequency ranges across the cap), and for adult imaging sessions also the quality of control word‐hearing task activation maps, as in prior studies (Eggebrecht et al., 2014). Light fall‐off curve assessments excluded measurements bridging the dorsal panel to the rest of the cap, as those source‐detector distances are especially sensitive to head size and placement of the dorsal panel. Sessions and runs with poor data quality based on all the above metrics were excluded from further analysis.

#### 2.6.6 Task data analysis

For the word‐hearing task, a general linear model (GLM) was used to calculate beta values of stimulus response relative to rest for each run in voxel space, using a canonical adult hemodynamic response function previously determined empirically using HD‐DOT (Hassanpour et al., 2014). A GLM was similarly used to analyze finger tapping data, but for this lateralized task, the contrast between left‐sided and right‐sided responses was mapped by calculating the difference in beta values between those two task conditions. As the continuous visual stimulation task was not conducive to an event‐design GLM analysis, responses were instead block‐averaged across a 6‐s time window beginning 7 s after the wedge had entered the lower left and lower right visual quadrants, based on prior studies using the same stimuli (Tripathy et al., 2021). The contrast between left‐sided and right‐sided visual responses was then mapped by calculating the difference in the hemoglobin signal response between those two stimulus conditions. Group‐level maps were generated by applying an affine transform to move subject‐specific images into MNI152 atlas space for each run and then calculating random effects t‐statistics across all runs from all participants in the group. Maps were finally plotted on an atlas surface. Group‐level response time courses were plotted by averaging responses in regions of interest across all blocks, runs, and participants. The region of interest was designated for each task by thresholding the group average activation map at 25% of the maximum t‐statistic value. Responses are relative to the baseline initial fixation period prior to task onset. Error bars represent the standard error of the mean across all blocks (standard deviation divided by the square root of the total number of blocks).

#### 2.6.7 Movie‐viewing data analysis

Precise timing for the start of the movie during each run is required for alignment between the movie runs and feature regressors. The start time of each movie was computed by calculating cross‐correlations between the movie audio collected during each imaging run (recorded with the HD‐DOT data through one of the ADC channels). Data time courses were then shifted by the lag that maximized the correlation between the collected and known audio signals extracted from the movie file. This approach corrected for any variable stimulus delays caused by the experimenter or equipment, allowing highly timing‐sensitive analyses like feature regression to be accurately time‐locked (Vanderwal et al., 2019).

To study inter‐run synchronization, voxel‐wise correlations were calculated between the oxyhemoglobin signals across time from the two movie runs of interest (Fishell et al., 2019; Golland et al., 2007; Hasson et al., 2004). Random effects *t*‐statistic maps were constructed to aggregate the results across multiple pairs of runs and allow comparisons between conditions, for example across all matched movie run pairs, or across mismatched movie run pairs as a negative control (Fishell et al., 2019; Sherafati et al., 2020).

For feature regressor analysis, speech and face regressors were coded for every movie clip in the library by three independent experimenters, using a protocol adapted from previous studies (Bartels & Zeki, 2004; Fishell et al., 2019). Speech content was scored on a binary basis (present or not) in 1‐s bins across each movie clip. The salience of faces in the scene was graded on a 4‐point scale (0 = absent, 1 = present but in the background, 2 = present and salient, 3 = filling the screen or otherwise highly salient) for frames sampled at 1‐s intervals across each movie clip. Each coded regressor was averaged across the three experimenter's ratings, with a final reviewer resolving major disagreements at any time points attributable to human error. While automatic approaches for detecting speech and faces were also considered, these techniques are built upon manually coded datasets and traditionally focused on real‐world face detection leading to inaccuracies when applied to the task of animated face detection (Kim et al., 2021). The resulting consensus regressors were then convolved with a canonical hemodynamic response function (Hassanpour et al., 2014) and *z*‐scored. In the current study, the same hemodynamic response function was used for both adult and child analysis. Both regressors and HD‐DOT data were filtered with the same high‐pass cutoff of 0.02 and low‐pass cutoff of 0.1 prior to correlation analysis.

To conduct univariate regressor analysis (e.g., simple speech mapping) for a run of data, voxel‐wise Pearson correlations $r_{v,f}$ were calculated across the run between the zero‐mean‐centered oxyhemoglobin signal timecourse $S_{v}$ for the vth voxel and the regressor $R_{f}$ for feature f, as in previous studies (Fishell et al., 2019):

(6) $$r_{v,f}=\frac{\left(S_{v}\right)∙\left(R_{f}\right)}{\left|S_{v}\right||R_{f}|}$$

For a more refined version of parallel feature mapping, voxel‐wise feature correlations were evaluated by calculating regression model coefficients using a multivariate linear regression approach. Multiple features of interest (speech and faces) and nuisance regressors (GVTD, audio envelope, luminance, temporal derivative of luminance, temporal derivative of squared luminance, hands, and bodies) were incorporated into a design matrix, X. The data Y were modeled mathematically as a function of X and a matrix of regression coefficients, β:

(7) Y=Xβ+e

where the error term e represents zero‐mean Gaussian noise. The pseudoinverse of X was then used to estimate β from the data through the method of least squares:

(8) $$\beta ={\left(X^{T}X\right)}^{-1}X^{T}Y$$

In all cases, results were aggregated across runs by calculating random effects *t*‐statistics and plotting them on an atlas surface.

To evaluate how much temporal covariance between feature regressors could drive the spatial overlap between regressor maps, we computed the correlation M between the zero‐mean‐centered speech and face maps, S and F, as well as the correlation R between the speech and face regressors, s and f, for all runs across all participants. For the ith run in our data set:

(9) $$M_{i}=\frac{\left(S_{i}\right)∙\left(F_{i}\right)}{\left|S_{i}\right||F_{i}|}$$

(10) $$R_{i}=\frac{\left(s_{i}\right)∙\left(f_{i}\right)}{\left|s_{i}\right|\left|f_{i}\right|}$$

### 2.7 Data and code sharing

To enable further analysis by other groups, our adult localizer and movie viewing data is available publicly through NITRC (https://www.nitrc.org/projects/neurodot) and the pediatric imaging data is available upon request by contacting the corresponding authors. Code for processing HD‐DOT data is publicly available through NITRC (https://www.nitrc.org/projects/neurodot), and additional code specific to movie‐viewing data analysis can also be obtained by contacting the authors.

## 3 RESULTS

### 3.1 HD‐DOT imaging system characterization

Our new HD‐DOT system incorporated design features to increase child comfort as well as various advancements to enhance image quality, including a higher channel count and optode density, expanded field of view, and enhanced signal levels relative to previous systems. (Eggebrecht et al., 2014; Fishell et al., 2020). The grid of 128 sources and 125 detectors with 11 mm spacing between neighboring optodes generated approximately 3445 measurements (source‐detector pairings) per wavelength in total (Figure 1a,b). However, with these high‐density systems, methodical preprocessing is necessary to select only measurements having meaningful signal‐to‐noise. We used a combination of source‐detector distance (<50 mm, ~2464 measurements) and measurement standard deviation (<7.5%) to categorize usable measurements. On average, 2237 measurements were retained for adults and 2051 measurements were retained for children. These measurements were distributed across the posterior, right and left lateral, and dorsal surfaces of the head (Figure 1c) to cover significant portions of occipital, temporal, and parietal cortex including visual, auditory, sensorimotor, and association regions (Figure 1d,e). While the cap does not cover all of the frontal cortex, it does include some important frontal regions as well, including primary motor cortex, dorsolateral prefrontal cortex, and Broca's area. To maximize the dynamic range of measurements, laser light sources were used with a “2‐pass encoding pattern”, wherein every source was modulated in turn with square waves with a duty cycle of first 1% and then 50%. As evidenced by data acquired using an optical phantom (head phantom with 57.5 cm circumference, Gower Labs), the 2‐pass encoding pattern yielded light fall‐off curves spanning seven orders of magnitude of optical power above the noise floor without clipping. These measurements spanned a wider range of light levels than those effectively obtained with any single intensity illumination (Figure 1f–h), and justified the choice of a 50 mm source‐detector separation cutoff for the flat‐field reconstructions for mapping sensitivity (Figure 1d). System features including sensitivity, detectivity, crosstalk, dynamic range, and frame rate (measured as detailed in Supplemental Methods) are summarized in Table 1. The goal values presented in Table 1 are based on previous system designs (Eggebrecht et al., 2014; Fishell et al., 2020).

**TABLE 1:** System characterization.

System specification	Goal	Actual (mean)
Sensitivity (V/W)	>1 × 10^6^	6 × 10^6^
Noise equivalent power (fW/√Hz)	<30	16.3
Optical noise floor (fW)	<1340	755
Detectivity (fW/[√Hz*mm^2^])	<350	10.6
Crosstalk (dB)	<−120	−131.5
Dynamic range (dB in 1 Hz)	>120	156.4
Frame rate (Hz)	>3	10.4

### 3.2 System validation in adults

**FIGURE 2:**
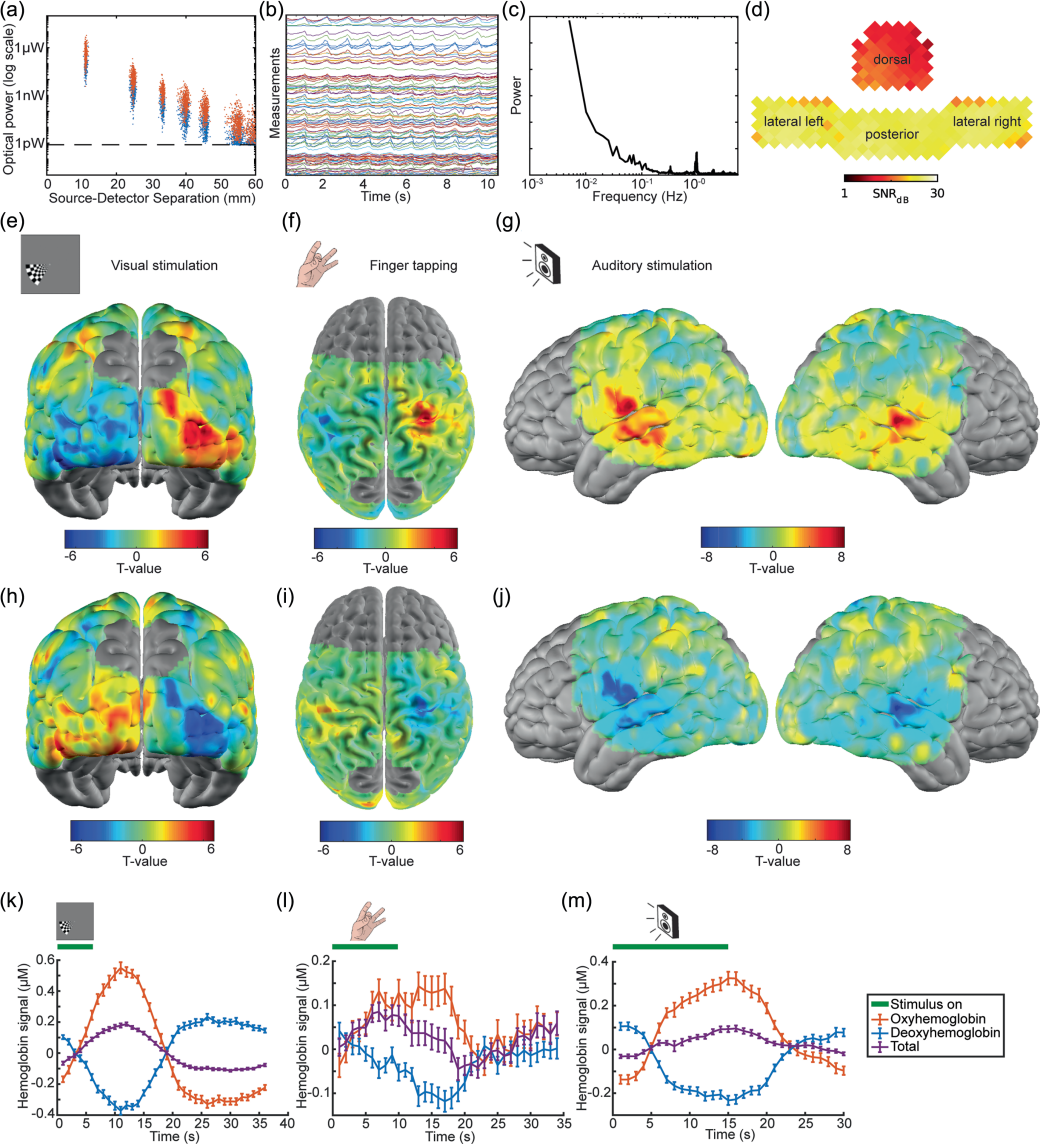
System validation with in vivo adult data: (a–d) Representative single subject data quality illustrations. (a) Light levels show a log‐linear fall‐off with increasing source‐detector separation. Optical power measurements are tightly clustered within 2 orders of magnitude at each possible distance and are above the noise floor across 5 degrees of source‐detector separation. (b) The cardiac pulse is clearly visible in measurement time traces. (c) Fourier spectra show a strong cardiac pulse peak at ~1 Hz. (d) High mean 0.5–2 Hz band‐limited signal‐to‐noise ratio (pulse SNR) is detectible across the cap. SNR tended to be higher across the posterior and lateral panels than the dorsal panel. (e–j) Group level oxyhemoglobin and deoxyhemoglobin maps from 5 adult participants illustrate image quality across the cap. OxyHb (e) and deoxyHb (h) contrast maps subtracting the response to right‐sided visual stimuli from the response to left‐sided visual stimuli illustrate robust contralateral visual cortex activations across 8 runs. OxyHb (f) and deoxyHb (i) contrast maps for left‐sided finger tapping minus right‐sided finger tapping show the contralateral motor cortex activity evoked during 9 runs of a motor task. Robust bilateral auditory cortex oxyHb (G) and deoxyHb (j) responses measured in response to 8 word‐hearing task runs. (k–m) Corresponding plots of oxy‐, deoxy‐, and total hemoglobin signals averaged across regions of activation in all participants.

In vivo data were first collected in adults to validate the performance of the new system using simple tasks in a compliant and well‐characterized reference population before pursuing studies of more complex paradigms like movie viewing or more challenging populations like children. We regularly observed a log‐linear fall‐off of light intensity with source‐detector separation, with clustering of measurements within ~2 orders of magnitude at each separation, and with measurements consistently above the noise floor out to at least fifth‐nearest neighbor pairs (Figure 2a). The cardiac pulse waveform was clearly visible in measurement time traces (Figure 2b) and a corresponding peak around 1 Hz was notable in Fourier spectra of measurements (Figure 2c). Plotting the ratio of signal power in the 0.5–2 Hz frequency band to the bandwidth‐scaled median power in flanking frequency ranges revealed high pulse SNR across the cap, although SNR was frequently higher across the lateral and posterior panels than the dorsal panel (Figure 2d). Task activation maps provided further evidence of image quality. Mapping the oxyhemoglobin signal, we detected contralateral visual cortex activations in response to unilateral checkerboard stimuli (Figure 2e), contralateral motor cortex activation during unilateral finger tapping (Figure 2f), and bilateral auditory cortex activations during a word‐hearing task (Figure 2g). Corresponding deoxyhemoglobin signal maps illustrated consistent results (Figure 2h–j). Oxyhemoglobin maps for individual subjects are included in Supplemental Figure S5A. We also report the corresponding time courses of oxyhemoglobin, deoxyhemoglobin, and total hemoglobin signals (Figure 2k–m).

### 3.3 Characterization of children's movie stimulus library in adults

**FIGURE 3:**
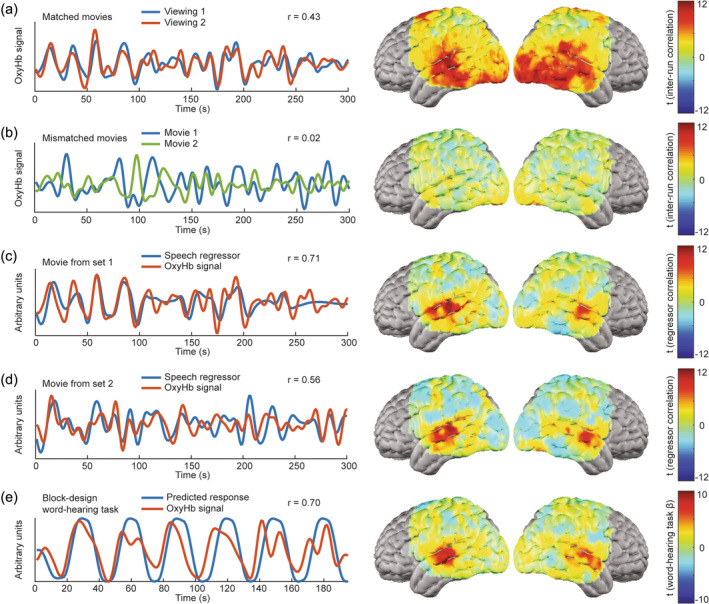
Functional brain mapping using animated movies: Movie data from 3 highly sampled adults (80 movie‐viewing runs total): Illustrative time traces are plotted for a voxel of interest in the superior temporal gyrus ([−67.5, −27,12]) in an individual participant, while group maps are presented to show results across voxels, runs, and participants. (a) Movies evoked reproducible patterns of brain activity, plotted here for a single voxel during two viewings of the same clip, and mapped across the brain as the t‐statistics for voxel‐wise inter‐run signal correlations over time for all 40 pairs of movie‐viewing runs. (b) These responses are movie‐specific – comparing responses across 40 pairs of mismatched movie clips does not reveal the same inter‐run synchronization seen with matched clips. (c) A heterogeneous set of movies can be used to map responses to movie features such as speech through regressor correlation analysis. An exemplary speech regressor time course convolved with a canonical hemodynamic response function is plotted here alongside the oxyhemoglobin signal in a voxel of the brain that appears to be responsive to speech. The adjacent group speech regressor map highlights regions putatively involved in speech processing across the field of view. The t‐statistics plotted here are calculated from voxel‐wise correlations with speech regressors across 32 movie‐viewing runs using 8 different clips from the movies Moana and Finding Nemo. (d) A second group speech regressor map is shown here from a separate set of 34 movie‐viewing runs using an entirely separate set of eight movie clips from Curious George and Frozen. Speech regressors may have different time courses for different movies, but feature regressor analysis produces maps that are comparable nonetheless. (e) Activation maps from 22 runs of session‐matched, block‐design, word‐hearing task data are comparable to movie‐derived speech regressor maps.

In order to develop more engaging, age‐appropriate stimuli for children, we compiled a library of animated movie clips and an associated data analysis pipeline. Movie viewing responses were analyzed across a total of 80 movie‐viewing task runs from three adult participants (Supplemental Table 1). Similarities in the cortical response between two independent viewings of a single clip were evident from time courses in individual regions of interest (illustrated in Figure 3 for a voxel from superior temporal gyrus). Mapping inter‐run correlations across the field of view, we captured reproducible responses to any given movie over independent viewings of the same clip (“matched movie runs”) across large swaths of temporal, parietal, and occipital cortex (Figure 3a). Mapping the correlations between responses to two different movie clips (i.e., “mismatched movies runs”) did not yield the same inter‐run synchronization (Figure 3b). However, any movie clip, or even a combination of movie clips, could be used to map responses to specific movie features through feature regressor analysis. For instance, we constructed speech regressor maps highlighting regions putatively involved in processing speech (Figure 3c,d). Speech regressor maps were similar across two completely independent sets of various movie clips (using clips from Moana and Finding Nemo in Figure 3c, and clips from Curious George and Frozen in Figure 3d). Speech regressor maps also appeared comparable to activation maps from conventional block‐design, word‐hearing task data (Figure 3e), which were collected between the movie viewing runs (22 session‐matched word‐hearing runs across the three participants).

**FIGURE 4:**
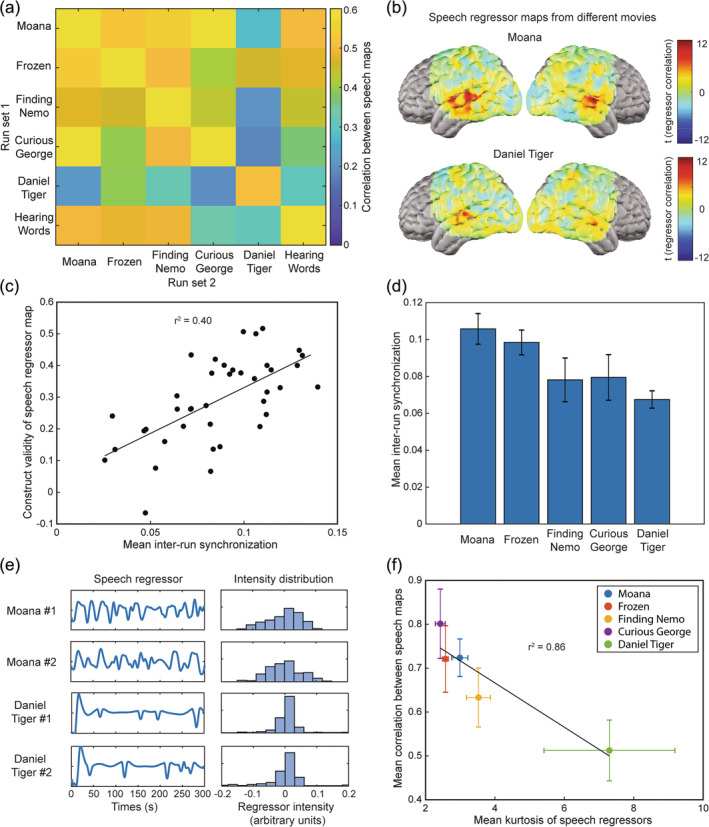
Comparison of regressor maps across movies: (a) Correlation matrix comparing speech regressor maps from participants viewing clips of five different children's movies/shows as well as word‐hearing task activation maps. High correlations between independent viewings from the same movie (along the main diagonal), between different movies (off the main diagonal) except for Daniel Tiger's Neighborhood, and with the word‐hearing task (final row and column) illustrate the overall reproducibility, generalizability, and construct validity of speech regressor analysis. (b) Regressor correlations are weaker and noisier for some movies (particularly Daniel Tiger's Neighborhood) compared to others (e.g., Moana). (c) The construct validity of the speech regressor maps from a movie clip (measured as the spatial correlation between the mean speech regressor map and a session‐matched word‐hearing task activation map) is positively correlated with the mean inter‐run synchronization of measured brain activity between independent viewings of the movie clip. In this graph, each data point represents one pair of matched movie runs (i.e., the same participant viewing the same movie twice), and all 40 matched movie run pairs across all three adults are plotted. (d) A bar graph comparing the mean inter‐run synchronization of measured brain activity in our data from different movies. The show Daniel Tiger's Neighborhood was associated with lower mean inter‐run synchronization than other movies. (e) Modulation of speech over time is lower for clips of Daniel Tiger's Neighborhood than the other movies (e.g., Moana), as illustrated by exemplary regressor time courses and corresponding histograms of the regressor intensity distribution across time points (in arbitrary units, scaled relative to the norm of the distribution). (f) Quantifying and extending results from panel E, the kurtosis of the regressor intensity distribution is higher for clips from Daniel Tiger's Neighborhood than for the other movies. The reproducibility of speech maps decreases with increasing kurtosis of the underlying speech regressors.

Capitalizing on the plurality of movie clips in our stimulus library, we further assessed potential factors influencing the quality of speech regressor maps. To evaluate whether the specific clip that was presented mattered for the map ultimately produced, we constructed two independent group‐level regressor maps, using two separate sets of movie‐viewing runs, for each source movie or television show. We then calculated spatial correlations between every possible pairing of the resulting regressor maps, measuring reproducibility (Figure 4a). We also calculated spatial correlations between each movie's speech regressor map and a reference word‐hearing task map as a measure of construct validity (Figure 4a). Across all but one of the movies, both reproducibility and construct validity were consistently high (mean ± standard deviation of spatial correlations = 0.56 ± 0.12 for reproducibility and 0.45 ± 0.06 for construct validity), the outlier being the show Daniel Tiger's Neighborhood. As a negative control, maps constructed from the same imaging data using speech regressors from intentionally mismatched movies did not exhibit the same consistency (reproducibility = 0.11 ± 0.12 and construct validity = 0.09 ± 0.11). Statistical significance of inter‐map correlations was established by using a paired t‐test to compare the correlation values from all the true speech regressor maps to those from the negative control mismatched regressor maps (*p* = 1.24 × 10^−10^ for reproducibility and *p* = 1.7 × 10^−5^ for construct validity). Regressor correlations were notably weaker and noisier for Daniel Tiger's Neighborhood than for any of the other movies (Figure 4b). We identified several potential sources of this variance. The inter‐run synchronization of responses between matched movie‐viewing runs (using the same movie stimulus in the same participant) was a positive predictor of the construct validity of speech maps from those runs (Figure 4c). Moreover, in our data, runs using clips from Daniel Tiger's Neighborhood displayed lower inter‐run synchronization than runs using other movies (Figure 4d). Furthermore, speech regressors for Daniel Tiger's Neighborhood also displayed less modulation over time than those for other movies (as illustrated in Figure 4e), an effect quantified by computing the kurtosis of the distribution of regressor intensity values for each movie clip. A clear association was seen between the reproducibility of speech maps and the kurtosis of the underlying speech regressors across movies (Figure 4f).

### 3.4 Parallel feature mapping with multivariate regression

**FIGURE 5:**
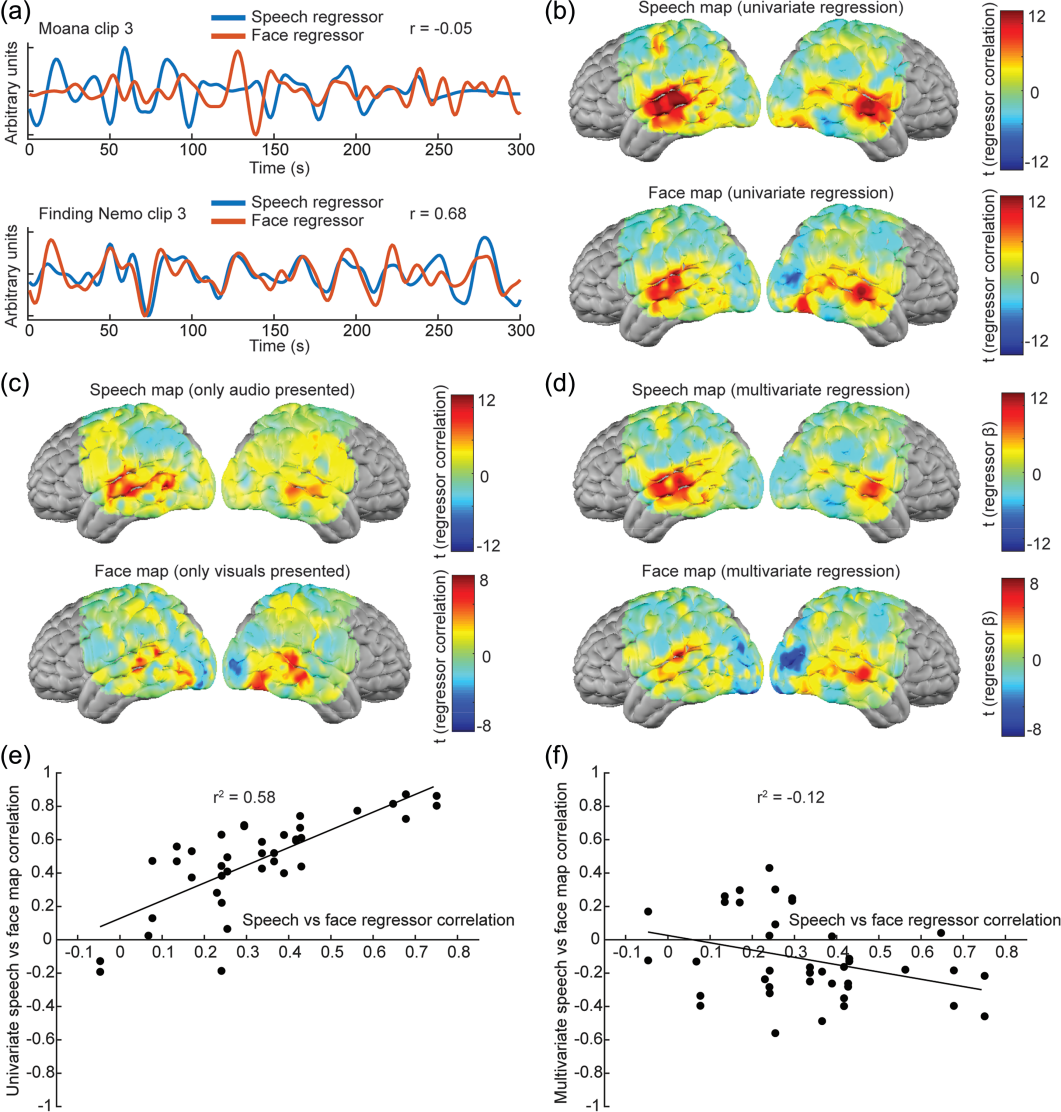
Parallel feature regressor mapping: (a) Because movies are such rich stimuli, movie‐viewing data can be used to map responses to multiple features of interest present in a movie, e.g. both speech and faces. However, these features of interest may be more independent for some movies (e.g., Moana clip 3) and more correlated with one another for other clips (e.g., Finding Nemo clip 3). (b) Perhaps as a result, feature correlation maps for speech and faces display notable overlap (t‐maps shown for regressor correlations from 80 movie viewing runs). (c) When a participant was presented with just the audio (containing speech and no faces; 16 runs) or just the visuals (containing faces and no speech; 10 runs) of movies to remove confounds, speech and face regressor maps appeared more distinct and lateralized. (d) Distinct speech and face maps could be obtained from the audiovisual movie‐viewing data through multivariate regression (t‐maps shown for regressor correlations from 80 movie viewing runs). (e) Using univariate regression, the overlap between speech and face maps for a movie was strongly correlated with the similarity of the speech and face regressors for that movie. (f) Multivariate regression abolished the strong positive correlation between regressor map overlap and regressor similarity.

Since movies are such rich, multi‐dimensional stimuli, they should be conducive to mapping multiple types of processing in parallel but may also result in confounding of responses to overlapping features (Bartels & Zeki, 2004; Fishell et al., 2019). To assess these considerations, we also manually coded face regressors for all our movies indicating the salience of faces across the clips. Since both speech and face regressors were manually coded, we assessed the interrater variability across all movie clips by computing the Pearson pairwise correlation between the individually rated regressors. We found an average correlation coefficient of 0.88 for the speech regressors and 0.78 for the face regressors. The additional variability in the face ratings reflects greater inter‐rater differences in scoring the salience of faces than in binary speech coding. However, all regressors were verified by a final rater to ensure regressor accuracy. For some clips, the time traces for the speech and face features appeared linearly independent, while for other clips they exhibited considerable covariance over time (Figure 5a). Face and speech regressor maps constructed from the same audiovisual movie‐viewing data using simple linear feature regression displayed notable overlap (Figure 5b). For comparison, we also computed speech regressor maps from a participant presented with movie audio unaccompanied by visuals, as well as face regressor maps from the same participant when presented with silent visual clips stripped of the audio. These deconvoluted speech and face response maps were more distinct, with the face maps notably more right‐lateralized and the speech maps more left‐lateralized (Figure 5c). In an attempt to mitigate confounding of speech and face regressor maps from the audiovisual movie‐viewing data by the covariance between these two movie features, we reran the analysis using a multivariate regression approach (as per Equations 7 and 8 in Section 2.6.7). This multivariate regression yielded more distinct and lateralized speech and face regressor maps (Figure 5d).

Across the regressor maps generated using simple linear regression, the similarity between face and speech maps (quantified by $M_{i}$ in Equation 9) and the similarity between face and speech regressors (quantified by $R_{i}$ in Equation 10) were strongly correlated (Figure 5e). However, the multivariate regression approach yielded more orthogonal maps and abolished the strong positive correlation between regressor map similarity and regressor similarity (Figure 5f).

### 3.5 Functional brain mapping in awake young children

**FIGURE 6:**
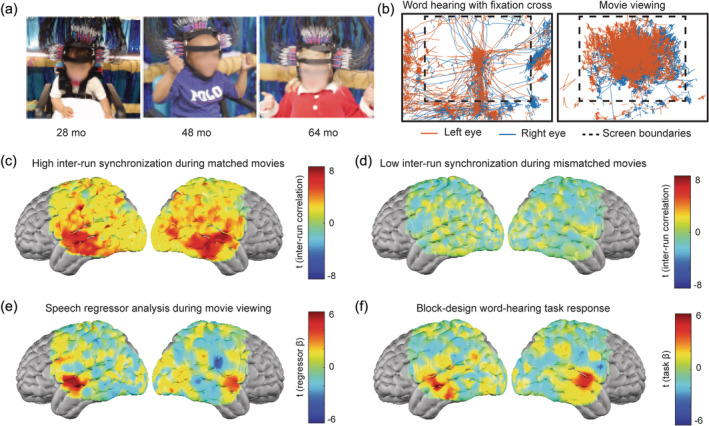
Functional brain mapping during movie viewing in young children using HD‐DOT: (a) *N* = 23 children ranging from 23 to 81 months of age were successfully imaged. (b) Eye tracking data showing a child attending to the stimulus display more when presented with movies than when asked to maintain visual fixation on a central crosshair. (c) High inter‐run synchronization is seen in responses to matched movie clips in the children across 56 movie‐viewing runs. (d) Evoked responses are movie‐specific, with low inter‐run synchronization across mismatched movie‐viewing runs. (e) Feature regressor analysis can be used to map receptive language from the HD‐DOT movie‐viewing data collected in awake young children. (f) Comparable responses are mapped across 17 block‐design word‐hearing task runs from children who complied with this task.

Having developed and validated the new imaging system, stimulus library, and data analysis pipelines, we sought to combine these tools to map responses to speech in young children. Having previously studied infants while asleep (Ferradal et al., 2016; Liao et al., 2012) and children older than 7 while awake (Fishell et al., 2020), we recruited children between 1 and 7 years of age and attempted to image them while awake (Figure 6a). Children appeared to be engaged when presented with a movie more than when asked to maintain central fixation during a conventional word‐hearing task, based on both experimenter observations as well as eye tracking data (Figure 6b). We obtained 56 usable movie‐viewing runs and 17 usable word‐hearing task runs from 23 children (Supplemental Table 2). Data quality was evaluated as described for adults in Sections 2.6.5 and 3.1, yielding comparable light fall‐off curves and pulse signals. (Supplemental Figure S6A–D). Although data quality was more variable in children with fewer measurements surviving preprocessing compared to adults, usable proportions of measurements were retained across five degrees of source‐detector separation (Supplemental Figure S6E). Mapping correlations between the oxyhemoglobin signal evoked by independent viewings of the same movie illustrated high inter‐run synchronization across our field of view, especially in temporal cortex (Figure 6c). Correlations between responses to mismatched movies were also computed as a negative control, indicating the specificity with which we were able to map movie‐evoked responses in children (Figure 6d). Speech regressor analysis yielded speech maps with higher correlations centered over the superior temporal gyrus bilaterally compared to the rest of the brain, but with stronger correlations in the left hemisphere (Figure 6e). Comparable activations in the superior temporal gyrus were also observed with the smaller number of successful block‐design word‐hearing task runs in the children who complied with this task (Figure 6f). Individual subject level word hearing maps are presented in Supplemental Figure S5B.

## 4 DISCUSSION

In summary, we developed an HD‐DOT imaging system targeted towards imaging young children and designed to enhance both image quality and participant comfort. In addition, we compiled a library of animated movies and associated data analysis pipelines for functional brain mapping with child‐friendly stimuli. After characterizing and optimizing the imaging system, stimulus library, and analysis methods in adults, we combined these tools to map responses to naturalistic speech in a pilot group of awake young children. We therein establish the utility of HD‐DOT imaging combined with a movie‐viewing paradigm for mapping brain function in early development.

### 4.1 Design and validation of HD‐DOT system

Human studies of brain development during the first years of life have been constrained by the challenges of pediatric imaging. Young children are often uncomfortable lying alone, awake, and still for an extended duration inside the dark, cramped, and loud bore of the MRI scanner (Raschle et al., 2012). As a result, studies of early brain development have typically imaged infants while asleep and have relied on resting state functional connectivity analysis to assess the functional architecture of the developing brain (Smyser et al., 2011). While powerful, this approach does not account for differences in functional networks between awake and sleep states (Brier et al., 2019; Mitra et al., 2017) and does not allow for investigation of active processing during diverse task states. Exciting recent advances in OPM‐MEG imaging technology have allowed for more child‐friendly, open imaging environments that have proven effective for imaging children of all ages during naturalistic tasks (Boto et al., 2022; Hill et al., 2019). FNIRS imaging caps can similarly be used in an open scanning environment. wherein pediatric participants can sit comfortably next to caregivers or even in their laps in a child‐friendly space (Lloyd‐Fox et al., 2013, 2017). FNIRS systems additionally offer greater portability and lower cost than alternative imaging methods like fMRI and MEG. Therefore, fNIRS is also conducive to imaging children and studying naturalistic behaviors ranging from natural speech to parent–child interactions (Butler et al., 2020; Czeszumski et al., 2020; Lloyd‐Fox et al., 2010). Recent advancements in fNIRS signal processing and system designs have allowed for substantial improvements in fNIRS image quality (Fantini & Sassaroli, 2020; Yücel et al., 2017). However, the sparse optode arrays traditionally used by fNIRS studies still reduce spatial resolution and accuracy of anatomical reconstructions of data relative to what could be accomplished with denser HD‐DOT arrays (Fishell et al., 2020; White & Culver, 2010). For example, developmental studies using fNIRS in atypical populations such as children with autism have shown fascinating early differences in lateralization during language processing tasks (Butler et al., 2020; Kawakubo et al., 2009; Yeung et al., 2019). However, the sparse imaging arrays limited reported findings to differences between relatively coarse regions of the head. Further studies with HD‐DOT could more precisely localize these differences between typical and atypical development, potentially enabling us to further pinpoint early neural correlates of autism.

The channel count of the system presented here – with approximately 2464 source‐detector measurements per wavelength within 5 cm of separation – is orders of magnitude higher than traditional fNIRS systems. Prior pediatric fNIRS research has relied on systems with 1 to 50 channels (Lloyd‐Fox et al., 2010), and even more recent studies using high‐density systems have mostly reported using 300–500 channels (Fishell et al., 2020; Frijia et al., 2021). Several prior HD‐DOT studies in adults described a ~ 1200 channel system (Eggebrecht et al., 2014; Fishell et al., 2019; Tripathy et al., 2021), but the current system still represents a significant expansion. Even after removing noisy measurements during pre‐processing, an average of 2237 and 2051 measurements were retained for adults and children, respectively. These numerous channels cover a wide field of view and are also packed at a high measurement density. Previous developmental optical neuroimaging studies have mostly used fNIRS caps covering more restricted areas of the head surface, e.g. with unilateral or bilateral coverage of only the sides of the head (Fishell et al., 2020; Frijia et al., 2021; Lloyd‐Fox et al., 2010). While that approach may suffice for mapping changes in targeted brain regions responding to simple stimuli, it is blind to effects outside the a priori region of interest and cannot be used to map distributed responses to complex stimuli. Larger HD‐DOT caps with posterior and lateral panels have been used to effectively map resting state networks, localized evoked responses to various visual and language tasks, and more dispersed responses to naturalistic multi‐sensory stimuli in adults (Eggebrecht et al., 2014; Fishell et al., 2019). The current system further expands on the occipital and temporal coverage of those wide‐field imaging cap designs with an additional panel covering the dorsal surface of the head and providing more somatomotor cortex coverage. To our knowledge, this altogether represents the largest reported coverage for a child‐friendly HD‐DOT system to date. Moreover, the 11 mm separation between adjacent sources and detectors is comparable to the 10–13 mm spacing reported for other high‐performing HD‐DOT systems (Eggebrecht et al., 2014; Frijia et al., 2021). Despite all these advances, the layout of the cap still excludes much of frontal cortex and portions of temporal and parietal cortex, considered crucial for working memory, multisensory integration, some aspects of language processing, and various other cognitive processes (Hertrich et al., 2020; Rohe & Noppeney, 2016). The cap does still cover areas of motor cortex, dorsolateral prefrontal cortex, and Broca's region, all involved in various aspects of language processing and speech production. The field‐of‐view limitations must be factored into the design and interpretation of developmental studies, but the current system still represents an improvement in cortical coverage over prior systems, and future systems could further expand channel count and modify cap layout to include presently omitted regions.

The laser sources and 2‐pass encoding pattern were designed to facilitate a wide dynamic range, integral to collecting measurements across a range of source‐detector separations to provide depth sensitivity and remove superficial signal contamination (Gregg et al., 2010). While the higher intensity illumination captured the longer‐range source‐detector pair measurements with high SNR, the lower intensity illumination ensured that short range measurements were also obtained without being saturated. The effectiveness of this approach was evident in the quality of the light‐fall‐off curves obtained with both optical phantoms and human participants, with measurements both unclipped and above the noise floor from 11 to 50 mm of source‐detector separation, and with light levels clustering within two orders of magnitude at each separation and spanning 7 orders of magnitude above the noise floor altogether (Figures 1h and 2a). The pulse waveforms evident in measurement time traces, the ~1‐Hz peak in Fourier spectra, and the pulse band‐limited SNR plots all further validated the high SNR of the system. Activation t‐statistic maps and hemoglobin time courses across a range of tasks provided a final level of validation by illustrating the ability of the system to capture statistically significant, stimulus‐evoked hemodynamic signals as expected across the field of view. Collectively, retinotopic mapping, finger tapping, and word‐hearing tasks elicited activations in visual, motor, and auditory areas across the posterior, dorsal, and lateral panels of the cap, consistent with prior HD‐DOT and fMRI studies (Eggebrecht et al., 2014; Newbold et al., 2020). The motor task maps and time courses exhibited more noise than the other maps (Figure 2), likely because the dorsal panel was the newest part of the cap. While there are gaps between the dorsal and lateral panels of the cap (Figure 1c), measurements between the panels are retained as long as source‐detector separation remains less than 50 mm. Responses mapped during the finger tapping task illustrate that the system is capable of effectively sampling frontoparietal regions such as somatomotor cortex. However, future system developments will aim to further improve coverage to avoid signal loss particularly amid anatomical variability between participants. The precise design and usage of the dorsal panel, including the spaces between panels and the management of hair on the top of the head, will remain a particular focus of optimization in subsequent iterations and applications of the system.

### 4.2 Functional brain mapping with movies

Young children are less likely to cooperate with paradigms involving extended periods of visual fixation and monotonous stimuli such as flickering checkerboards and spoken word lists. Movies produced for entertainment, while more complex, are more engaging stimuli, increasing compliance and reducing head motion in children (Greene et al., 2018). Furthermore, despite their complexity, movies have been shown to evoke reproducible responses in the brain between independent viewings by several fMRI studies (Golland et al., 2007; Hasson et al., 2004; Hasson et al., 2010) and a recent HD‐DOT study in adults (Fishell et al., 2019). Here, we first replicated this result using our new HD‐DOT system and a library of animated children's movie clips (Figure 3a,b). Consistent with prior studies, we identified high inter‐run synchronization across broad regions of the cortex spanning visual, auditory, and association areas (Golland et al., 2007; Hasson et al., 2004). Our ability to map reproducible and specific responses to complex stimuli across the field of view provides another layer of evidence for the image quality of our new system.

Feature regressor analysis has also previously been used with neuroimaging data to map activity correlated with specific features contained in a movie (Bartels & Zeki, 2004; Fishell et al., 2019). Here, we not only observed similar responses to speech and faces as these prior studies, but also used our movie library to compare regressor maps across different clips. There have only been limited previous studies comparing functional brain maps across different movies. The overall consistency of regressor maps with notable exceptions for just a few movie clips is similar to observations with resting state functional connectivity analysis across different movies in prior fMRI research (Vanderwal et al., 2019). The similarity of speech regressor maps from independent viewings of the same movies (along the diagonal in Figure 4a) as well as using completely different mixtures of movie clips (Figure 3c,d and off‐diagonal results in Figure 4a) illustrates the overall reproducibility and generalizability of our results. Furthermore, the high similarity of speech regressor maps to maps from a block‐design word‐hearing task (Figure 3e and the final row and column of the matrix in Figure 4a) provide an additional indicator of construct validity for movie‐based speech regressor analysis. While our word‐hearing task provides a relatively simplistic reference for construct validity, using this previously well‐validated task offers a useful starting point, and future studies could compare tasks discerning the phonological, syntactic, and semantic layers of language processing. Altogether, the fact that we can use data from participants viewing various movie clips to obtain similar, meaningful regressor maps highlights the flexibility of the movie‐viewing paradigm for functional brain mapping and has major practical significance. We could, for instance, use different movies to study different age groups or cater to any individual child's movie preferences and maximize compliance without compromising feature regressor maps.

A closer look at differences across runs and across clips does, however, point to some interesting sources of variance. For example, the construct validity of speech regressor maps from a particular clip and participant was positively correlated with the inter‐run synchronization between the two viewings of that clip by the participant (Figure 4c). This finding appears intuitive: more consistent responses to a movie in general might be expected to yield more consistent and meaningful feature maps as well, as the participant is then more likely attending to the same movie features driving their brain activity across viewings. Moreover, while regressor maps were highly consistent for most of the movies used, clips from the show Daniel Tiger's Neighborhood appeared to yield lower quality speech maps that were less similar to other speech regressor maps or word‐hearing task maps (Figure 4a,b). One possible explanation is that clips of this show were also associated with lower inter‐run synchronization in the adult data (Figure 4d), perhaps linked to subjective reports by adult participants that these clips were less engaging than the other movies. However, another possible source of variance is the audiovisual content and time courses of the clips. The speech regressors for Daniel Tiger's Neighborhood exhibited less modulation in feature intensity than other movies (Figure 4e,f), which could help intuitively explain the less robust speech response maps. Regressor modulation should hence be evaluated during stimulus selection for future studies performing feature regressor mapping.

The richness of movies as a stimulus can be an asset by engaging participants and allowing experimenters to study the processing of multiple features in parallel. However, our parallel feature regressor analysis of speech and face responses sheds light on important associated considerations in the selection of stimuli, method of analysis, and interpretation of results. Using simple linear feature regression, our speech and face regressor maps had notable overlap, featuring prominent correlations in the superior temporal gyrus bilaterally (Figure 5b). There are several possible explanations for this result. It could represent overlapping neural processing pathways for two features that are commonly concurrently processed in social interactions – we often attend to people's faces when they are speaking (Bartels & Zeki, 2004; Calvert et al., 1997). Alternatively, the observed regressor maps could be signatures of distinct face and speech processing areas that are located too close to one another to be distinguished given the point‐spread function associated with the optics of the current system. For example, the primary and secondary auditory cortex lie in close proximity to the superior temporal sulcus that has been implicated in face processing (Tsao & Livingstone, 2008). However, we could also be observing an artifact of the stimuli used – faces and speech are often concurrently emphasized in movies, and this covariation could confound both maps (Figure 5a). Consistent with this hypothesis, we found that the similarity between face and speech maps generated from a movie clip was strongly predicted by the correlation between the face and speech regressors for that clip (Figure 5e). Future studies using movies to investigate processing of specific features of interest such as speech and faces should aim to select clips for which these regressors are more orthogonal, and other potentially salient and covarying features should also be considered and controlled as much as possible.

In the absence of linearly independent features, we reasoned that taking a multivariate approach to regressor analysis should mitigate confounding of results. This approach has been effective in previous fMRI studies of movie feature response mapping (Bartels & Zeki, 2004), but had not been used in previous HD‐DOT studies using movie‐viewing paradigms (Fishell et al., 2019). Indeed, we found that incorporating both features of interest and additional confounds of concern as regressors in the design matrix of a GLM yielded more distinct speech and face maps (Figure 5d), with speech map versus face map correlations more evenly distributed around zero and no longer strongly driven by speech and face regressor correlations (Figure 5f). The speech maps from the multivariate regression featured significant correlations in the superior temporal gyrus bilaterally that were stronger in the left hemisphere. Meanwhile, face maps were right‐lateralized with two more posteriorly located temporal hotspots than the speech maps. For comparison, left‐lateralized speech maps were also observed when a participant was presented with movie audio alone (i.e., with no confounding visual features), and right‐lateralized face regressor maps were also obtained when the participant was presented with movie visuals stripped of audio (Figure 5c). Furthermore, prior fMRI studies have similarly described predominantly left hemispheric specialization of speech processing (Cai et al., 2013; Knecht et al., 2000) and right hemispheric specialization of face processing, with face responsive areas including the posterior superior temporal sulcus, fusiform face area, and lateral occipital face area (Bartels & Zeki, 2004; Le Grand et al., 2003; Tsao & Livingstone, 2008). These results provide compelling evidence for the benefits of the multivariate regression approach for feature mapping: feature responses can be deconvoluted even with complex, multi‐dimensional stimuli and even if children request to watch specific movies that may not have been filmed with orthogonal features of interest. Future studies will be needed to evaluate what comprises a truly sufficient set of regressors for multivariate analysis, to address not only other features of interest but also other potential confounding features.

### 4.3 Imaging in awake young children

Finally, we used the imaging system, stimuli, and analysis pipelines that we developed and characterized in adults to study a group of children. It was apparent to the experimenters running imaging sessions that free viewing of movies was a more engaging task for the children that increased their compliance relative to the word‐hearing task requiring central fixation. Eye tracking provided further objective behavioral evidence for this claim (Figure 6b). There is a wealth of literature using eye tracking during various tasks as a tool to understand typical and atypical brain function and behavior (Black et al., 2017; Constantino et al., 2017). Future studies could further factor gaze position into visual feature regressor analysis, evaluate how brain activity measured with HD‐DOT and eye tracking data relate in awake young children during movie viewing or social interactions, and assess differences in populations such as children with autism.

As in adults, we were able to map high inter‐run synchronization between independent viewings of matched but not mismatched movie clips in children, showing that reproducible and specific responses to complex, multi‐sensory movie stimuli can already be found early in development (Figure 6c,d). Numerous fMRI studies have shown the reproducibility of movie‐evoked brain responses in older children and adults, but very few have evaluated this in children younger than 7 years (Long et al., 2017; Moraczewski et al., 2018; Richardson et al., 2018; Vanderwal et al., 2019). Inter‐run synchronization could itself be an important feature of interest when studying neurodevelopmental disorders (Sokolowski & Levine, 2023).

Through movie feature regressor analysis, we further mapped responses to speech in the superior temporal gyrus that were present bilaterally but stronger in the left hemisphere, showing that this adult‐like response pattern is also established early in development (Figure 6e). Despite dramatic behavioral changes in language reception and production in the first few years of life, fMRI research on language development has generally studied older children (Weiss‐Croft & Baldeweg, 2015). Limited prior work in young children has studied passive speech perception during sleep in 2–3 year‐olds (Redcay et al., 2008), focused on infants in the first year of life (Dehaene‐lambertz et al., 2002; Frijia et al., 2021), or used fNIRS arrays with low channel counts limiting image quality (Butler et al., 2020). Here, we utilized the child‐friendly HD‐DOT imaging environment and movie paradigm to image responses to naturalistic speech in awake children. While the current study was not sufficiently powered to make quantitative comparisons between age groups and task states, future studies could apply the methods developed herein with larger sample sizes to investigate developmental neuroscience questions about the processing of language and other socially relevant stimuli like faces. Participant and data retention are likely to increase with experience imaging young children, adjustments to improve comfort, and the gradual development of a wireless pediatric imaging system.

Future studies could also further refine methods to enhance image quality for pediatric populations. For example, the current study uses a generic head model based on a single adult participant with a smaller‐than‐average head circumference. Ideally, an individual‐specific head model would be generated for each participant in the study. However, this is not always feasible, especially when working with young children who may not be comfortable enough in an MRI scanner to provide the requisite anatomical images. In such situations, an atlas‐based approach could provide a valuable approximation (Ferradal et al., 2014), using age‐specific atlases to refine age‐specific head models. In future studies, photogrammetric approaches could be used to further optimize reconstructions that mitigate inter‐individual variability in anatomy and cap placement within age groups. These elaborate efforts were considered beyond the scope of the current study, as we were focused on determining the feasibility of imaging young children with HD‐DOT and not yet at the point of making conclusive comparative claims regarding different populations. Age‐specific head modeling will be a focus of future work that will facilitate larger studies and comparative analyses in children. On a similar note, we used a canonical adult hemodynamic response function (HRF) for all our GLM‐based analyses, but studies have shown potential differences in neurovascular coupling and the HRF in early development (Arichi et al., 2012; Blasi et al., 2011; Richter & Richter, 2003). Age‐specific HRFs could hence further optimize analyses, but developing these tools across the age range of our participants was beyond the scope of this paper and will be the focus of future work. With larger datasets and expanded tools, future work could further elaborate and validate some of the analyses from our adult data (e.g. regarding reproducibility and construct validity of movie‐derived functional maps) in children.

### 4.4 Future directions

Our pilot sample grouped together children across a 5‐year age range in order to establish imaging feasibility, but there is much variability in behavior and hence likely differences in brain function across this broad time span. Future studies with more participants at specific ages could further investigate longitudinal changes across early development, with a particular focus on 1–4 year‐olds who have been especially challenging to study with fMRI. We coded regressors to study processing of speech and faces, but other regressors could be coded for the same stimuli to study other behaviors of interest (e.g., basic visual processing, emotional responses, etc) using the same data. Automated approaches for feature coding using machine learning can be implemented for future studies to extract features without the influence of manual rating. Future work could also explore other movie‐based analyses such as inter‐subject synchronization, functional connectivity, and inter‐subject functional connectivity to compare other aspects of functional brain organization between children at different ages and adults (Hasson et al., 2004; Ren et al., 2017; Vanderwal et al., 2019). Furthermore, while movie viewing may be a powerful, child‐friendly paradigm for studying various perceptual processes, other experimental paradigms should also be combined with HD‐DOT imaging in order to investigate the developmental neurobiology of speech production (Butler et al., 2020), social processing (Lloyd‐Fox et al., 2013, 2017), parent–child interactions (Czeszumski et al., 2020), and various other behaviors.

These approaches could also be applied to better understand atypical development and plasticity. The early emergence of atypical speech and face processing could be characterized in children diagnosed with autism spectrum disorder as well as pre‐symptomatic at‐risk siblings (Costanzo et al., 2015). Effects of malnutrition on brain function could also be studied during the first 1000 days of life emphasized to be critical for brain development (Cusick & Georgieff, 2016), with low‐cost, portable imaging systems already shown to be feasible for use in low‐resource settings (Fishell et al., 2020). Further essential optimization of HD‐DOT imaging is ongoing – caps are being made wireless and more wearable (Zhao & Cooper, 2017), while algorithms to improve data quality through measures like motion processing and head modeling are being continually refined (Sherafati et al., 2020). Our work here, developing imaging tools and establishing the feasibility of mapping brain function in awake young children with HD‐DOT, lays an important foundation for future advances in developmental neuroscience with major potential clinical impact.

## AUTHOR CONTRIBUTIONS

Kalyan Tripathy: conceptualization, methodology, software, validation, formal analysis, investigation, resources, data curation, visualization, writing (original draft, review and editing), project administration, funding acquisition. Morgan Fogarty: methodology, software, validation, formal analysis, investigation, data curation, visualization, and writing (review and editing). Alexandra Svoboda: software, formal analysis, investigation, resources, data curation, visualization, writing (review and editing), and project administration. Mariel Schroeder: formal analysis, investigation, resources, data curation, visualization, writing (review and editing), and project administration. Sean Rafferty: methodology, validation, investigation, resources, data curation, and writing (review and editing). Edward Richter: conceptualization, methodology, software, validation, resources, project administration, and writing (review and editing). Christopher Tracy: methodology, resources. Patricia Mansfield: investigation, resources, data curation, and writing (review and editing). Madison Booth: resources. Andrew Fishell: conceptualization, methodology, software, and funding acquisition. Arefeh Sherafati: software, resources, and writing (review and editing). Zachary Markow: software and resources. Muriah Wheelock: methodology, resources, and writing (review and editing). Bradley Schlaggar: conceptualization, writing (review and editing), and funding acquisition. Ana María Arbeláez: conceptualization, writing (review and editing), funding acquisition. Christopher D. Smyser: conceptualization, methodology, resources, writing (review and editing), supervision, funding acquisition. Adam Eggebrecht: conceptualization, methodology, software, resources, visualization, writing (review and editing), supervision, funding acquisition. Joseph Culver: conceptualization, methodology, resources, visualization, writing (review and editing), supervision, funding acquisition.

## CONFLICT OF INTEREST STATEMENT

The authors declare no conflicts of interest.

## Supporting information


**Data S1.** Supporting information.

## Data Availability

To enable further analysis by other groups, our adult localizer and movie viewing data is available publicly through NITRC (https://www.nitrc.org/projects/neurodot) and the pediatric imaging data is available upon request by contacting the corresponding author.
