# Supplementary material for: Mapping brain function in adults and young children during naturalistic viewing with high‐density diffuse optical tomography
Source: Hum Brain Mapp. 2024 May 4;45(7):e26684. doi: 10.1002/hbm.26684 (PMC11069306; doi:10.1002/hbm.26684)
Supplement: Supplementary file 1 — Data S1. Supporting information. [file HBM-45-e26684-s001.docx]

**Supplemental Methods**

The avalanche photo diodes (APDs) were characterized by measuring their sensitivity, noise floor, noise equivalent power (NEP), and dynamic range (Table 1). These values were calculated as follows. First, the optical power of the light entering the APD ($P_{opt}$) and the RMS voltage of the APD ($V_{RMS}$) were determined when the light source was at 20 kHz with a 50% duty cycle square wave. The APD was then turned off and again the output of the APD was recorded. From this data, we computed the fast Fourier transform of one second of data while the APD was on (${FFT}_{sig}$), and one second of data while the APD was off (${FFT}_{dark}$).

Sensitivity is the electrical output (in Volts) per power of radiative optical input (in Watts) to the device (Equation S1). The noise voltage density is the latent output of the APD (Volts) with no input signal, which is computed using Equation S2. The ratio of the noise voltage density to the sensitivity provides the NEP (Equation S3), or the level of radiative input optical power which produces an output with a signal-to-noise ratio equal to 1. The input-referred optical noise floor can subsequently be calculated factoring in the 2143 Hz measurement bandwidth (Equation S4).

$$\left( S1 \right) Sensitivity= \frac{V_{RMS}}{P_{opt}}$$

$$\left( S2 \right) Noise Voltage Density= V_{RMS}*\left( \frac{{FFT}_{dark}}{{FFT}_{sig}} \right)$$

$$\left( S3 \right) NEP= \frac{Noise Voltage Density}{Sensitivity}$$

$$\left( S4 \right) Optical noise floor= NEP* \sqrt{Measurement bandwidth}$$

From here, detectivity, crosstalk, and dynamic range could be calculated. As per Equation S5, detectivity is the ratio of the NEP and the area of the detector (*Darea*), which in our case was taken as half of the area of the fiber bundle due to the space between individual fibers. The dynamic range (in dB) is computed using the maximum voltage of the APDs ($V_{max}$), the sensitivity, and the NEP (Equation S6).

$$\left( S5 \right) Detectivity= \frac{NEP}{Darea}$$

$$\left( S6 \right) Dynamic Range= 20*log(10)\left( \frac{\left( \frac{\frac{V_{max}}{\sqrt{2}}}{Sensitivity} \right)}{NEP} \right)$$

Finally, crosstalk is the output of an APD with no input signal to the output of an APD with a signal, recorded simultaneously (Equation S7). Measurements were collected for each APD in the box and the FFTs were computed as described above.

$${(S7) Crosstalk}_{i,j}=\frac{{FFT}_{i,dark}}{{FFT}_{j,sig}}$$

To determine the frame rate of the system, the number of samples per timestep was first computed by dividing the total number of samples by the number of samples collected per second. In our case, 144 samples were collected at 96000 samples per second. We then computed the number of samples per frame factoring in the number of timesteps and the 2-pass encoding pattern (Equation S8).

$$\left( S8 \right) Frame Rate= \frac{1}{\left( \frac{\# of Samples}{Sampling rate}*\#of timesteps \right)*\# of passes in encoding pattern}$$

**Supplemental Table ST1: Data Collection in Adults.**

|  | Number of runs during system validation sessions: | | | Number of runs during movie viewing sessions: | |
| --- | --- | --- | --- | --- | --- |
| Subject | Word hearing | Finger tapping | Retinotopy | Word hearing | Movie viewing |
| A01 | 2 | 2 | 1 | 6 | 18 |
| A02 | 2 | 2 | 2 | 0 | 0 |
| A03 | 1 | 1 | 1 | 6 | 22 |
| A04 | 1 | 2 | 2 | 10 | 40 |
| A05 | 2 | 2 | 2 | 0 | 0 |
| Total | 8 | 9 | 8 | 22 | 80 |

**Supplemental Table ST2: Data Collection for Children**

| Subject | Age (months) | Number of Movie-viewing runs | Number of Word-hearing runs |
| --- | --- | --- | --- |
| C01 | 23 | 4 | 0 |
| C02 | 28 | 5 | 1 |
| C03 | 42 | 2 | 0 |
| C04 | 43 | 3 | 1 |
| C05 | 48 | 4 | 1 |
| C06 | 48 | 1 | 0 |
| C07 | 49 | 8 | 2 |
| C08 | 49 | 2 | 0 |
| C09 | 50 | 3 | 0 |
| C10 | 54 | 3 | 0 |
| C11 | 54 | 2 | 0 |
| C12 | 59 | 3 | 3 |
| C13 | 61 | 1 | 0 |
| C14 | 67 | 0 | 2 |
| C15 | 67 | 0 | 1 |
| C16 | 67 | 1 | 0 |
| C17 | 70 | 6 | 2 |
| C18 | 70 | 0 | 1 |
| C19 | 70 | 0 | 1 |
| C20 | 75 | 4 | 0 |
| C21 | 75 | 1 | 0 |
| C22 | 76 | 3 | 1 |
| C23 | 81 | 0 | 1 |
| Total |  | 56 | 17 |

| 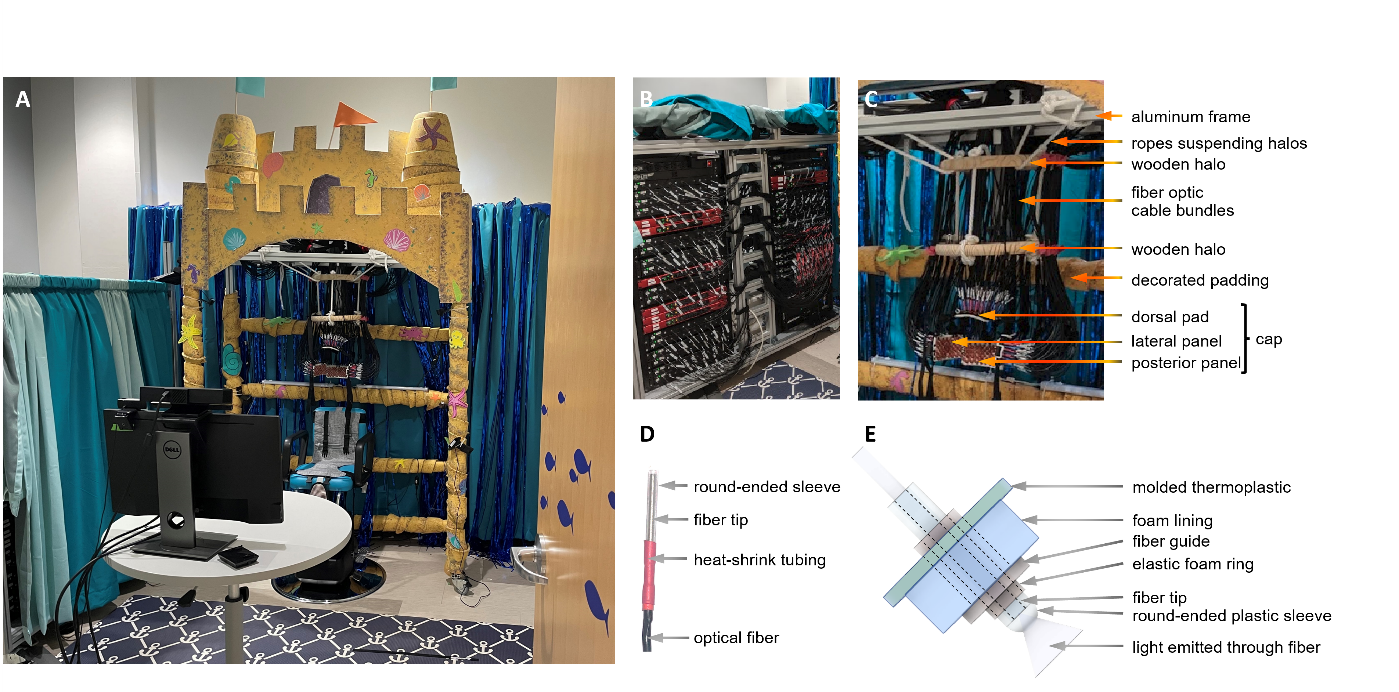 |
| --- |
| **Supplemental Figure S1: Preschooler HD-DOT imaging system design details**: (A) Child-friendly imaging suite. (B) Concealed behind curtains are stacks of sources, detectors, ADCs, and the source and detector computers, all on a mobile extruded aluminum cart. (C) The imaging cap is suspended from a mobile, decorated frame, with the weight and torque of optical fibers distributed symmetrically around a pair of halos and an extruded aluminum frame. (D) Fiber tips are capped with a round-ended soft plastic sleeve, secured in place by heat-shrink tubing. (E) Layers and components of the cap holding each of the 253 optical fiber tips in place around the head. |

| 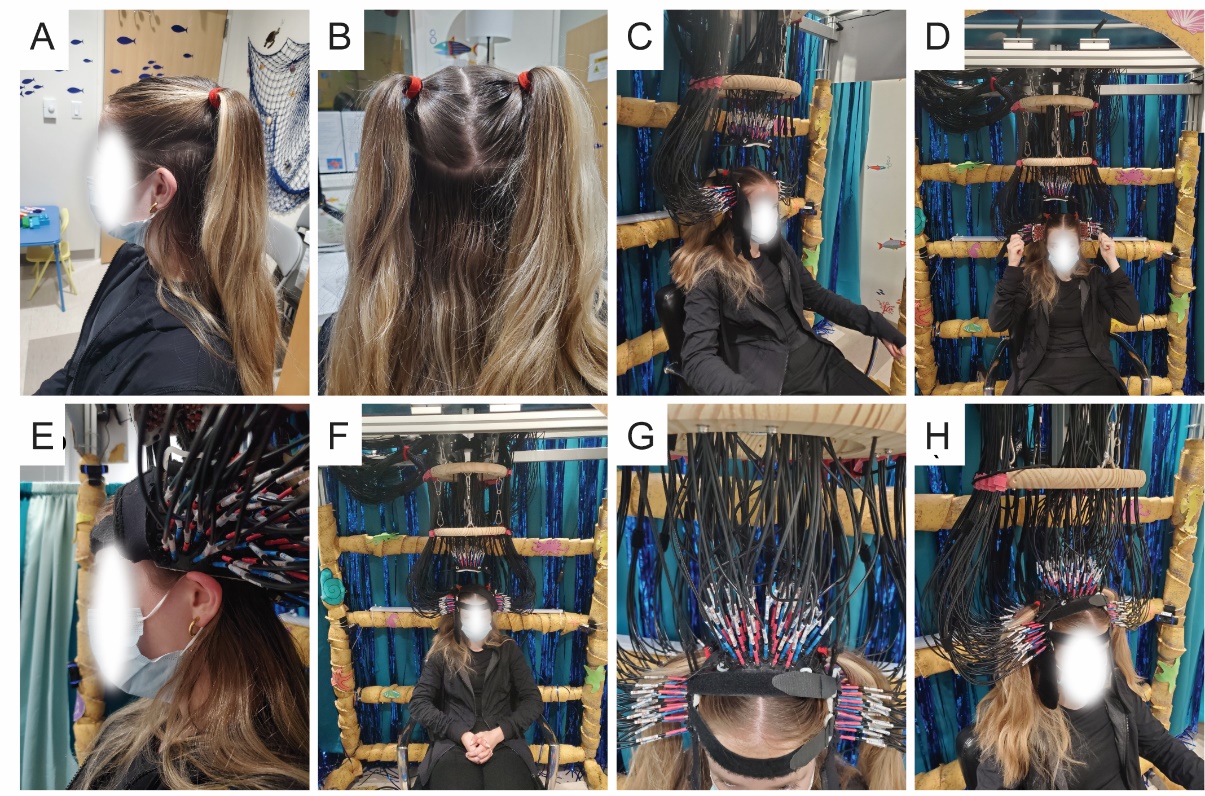 |
| --- |
| **Supplemental Figure S2: Cap fit procedure:** For participants with long hair, their hair is parted down the middle and back, then tied in left and right pigtails (A-B) to separate the hair for easy combing of the optodes. The participant then sits in the imaging chair and slides their head back into the cap (C) and the pigtails (if present) are threaded through the lateral and dorsal cap panels. The chair height is adjusted to position the participant’s ears just below the lateral panels of the cap. The participant then holds a pair of Velcro straps and is asked to move the cap and their head side-to-side to comb the optodes through their hair and onto the surface of the scalp (D). The tragus and fiducial markers on the cap are used as reference points to ensure the cap is positioned symmetrically and consistently, and Velcro straps are used to hold the cap onto the head (E-F). The dorsal imaging pad is lowered by an experimenter, combed through the hair, centered, and aligned with the rest of the cap before being strapped down (G-H). |
| 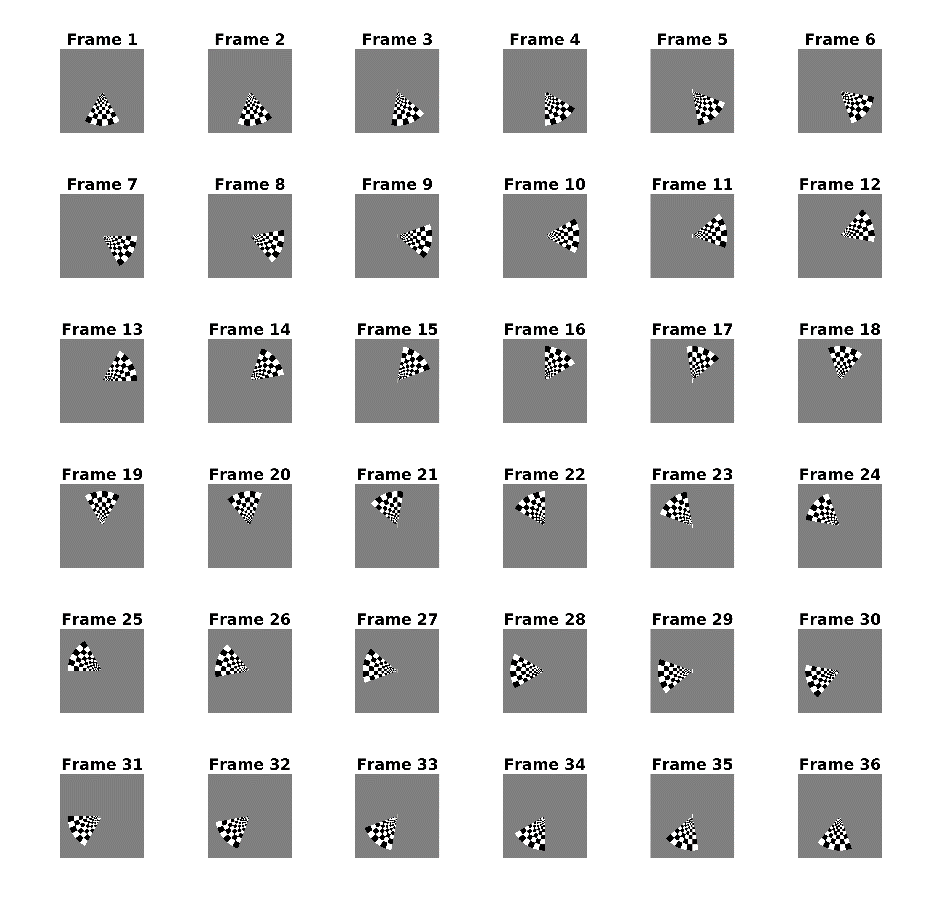 |
| **Supplemental Figure S3: Checkerboard stimuli**: A wedge-shaped black and white checkerboard pattern reversed at 8 Hz against a constant 50% grey background while rotating around the screen. The wedge subtended a polar angle of 60⁰, a radial angle from 2.5⁰ to 10⁰, and rotated 10⁰ at a time through 36 positions spanning 360⁰ at 1 second per position, for a total of 10 cycles per run. |

| 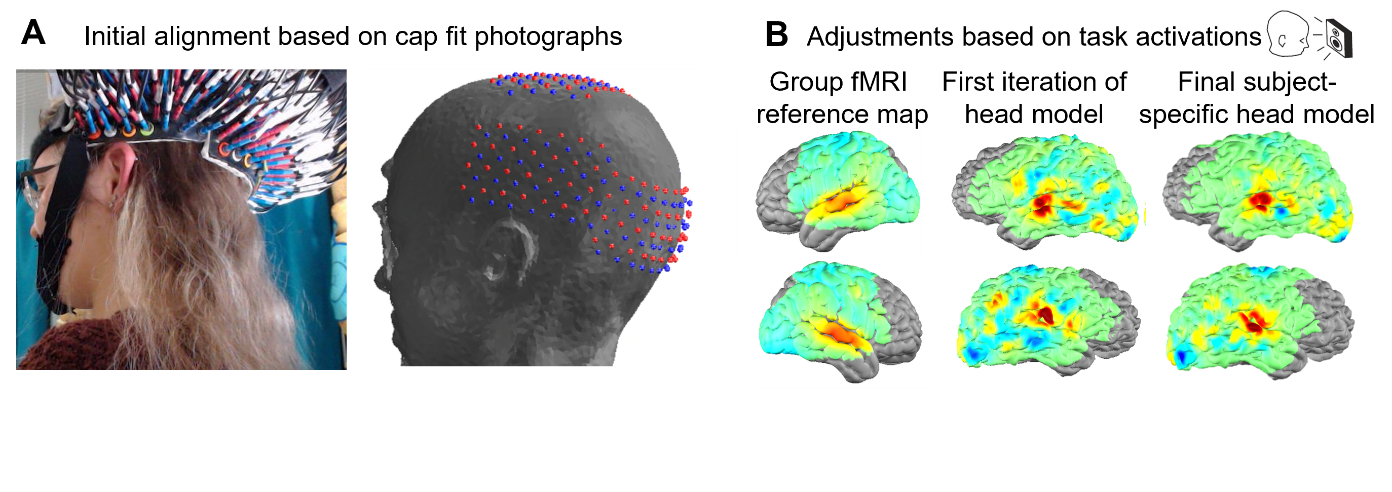 |
| --- |
| **Supplemental Figure S4: Subject-specific head modeling**: for optimized reconstructions of adult HD-DOT movie-viewing data: (A) Cap fit photographs taken from 7 camera angles were used to guide positioning of the optode array on a mesh of the participant’s head (made from the individual’s structural MRI data) for the first iteration of a subject-specific head model. (B) The participant’s word-hearing task HD-DOT data were used to guide adjustments to the head model based on how the reconstructed data compared to a reference group fMRI word-hearing task activation map. |

| 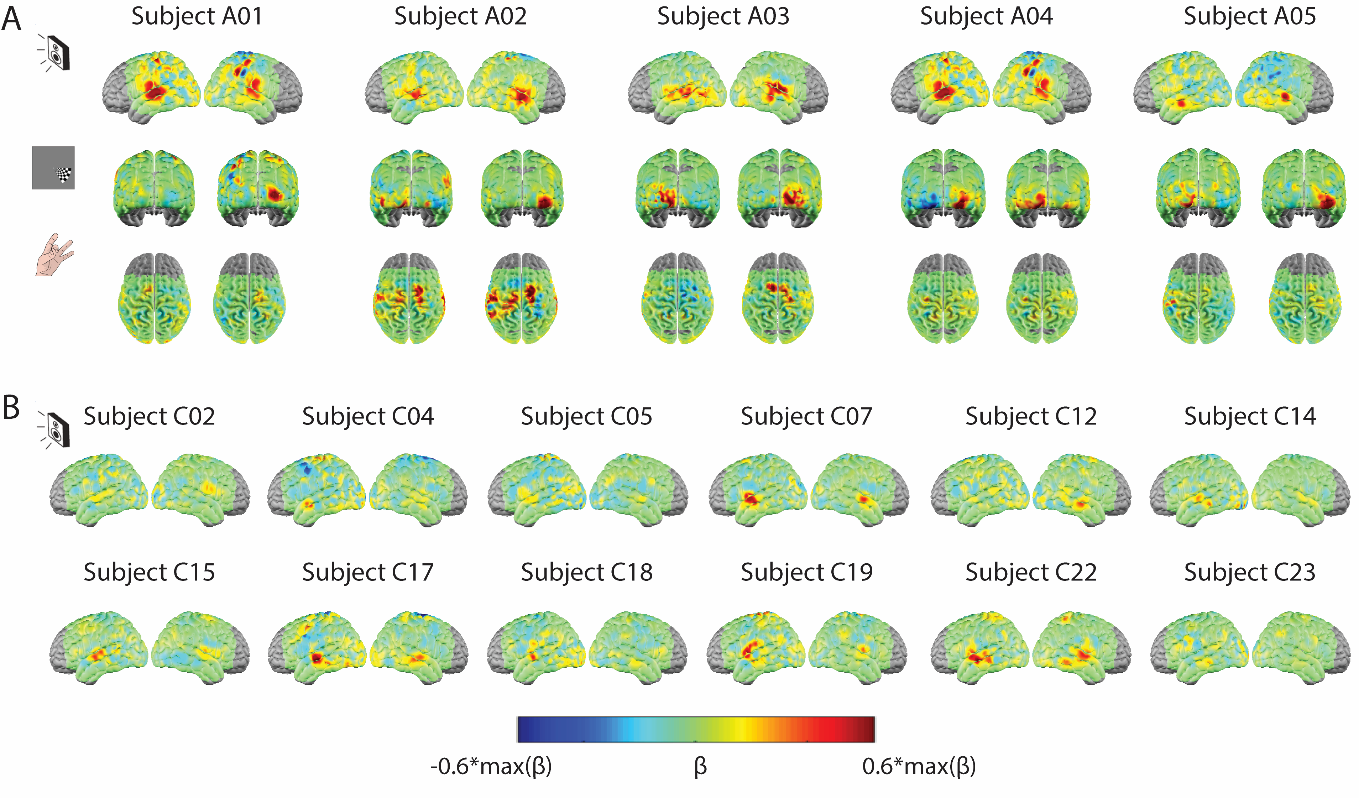 |
| --- |
| **Supplemental Figure S5: Individual maps**: Beta value maps were generated for individual task runs in each adult (A) and child (B) participant. Adult participants performed three conventional tasks: word hearing (top), retinotopy (middle), and finger tapping (bottom). Word hearing was the only conventional task done with children (B). Maps from one run of each task are shown for each participant to illustrate the range of signal variability across participants, sessions, and runs. |

| 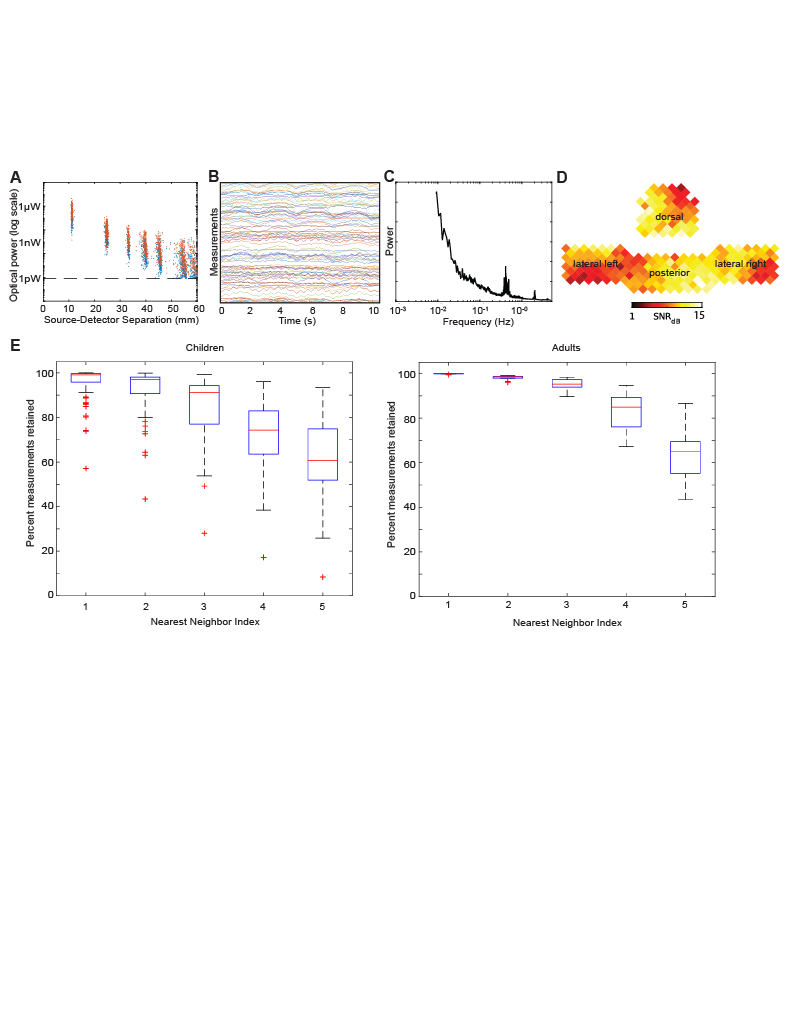 |
| --- |
| **Supplemental Figure S6: Pediatric data quality:** (A-D) Single subject data quality illustrations from one of the pediatric imaging sessions (A) Light fall-off plot showing the log-linear fall-off of light levels with increasing source detector distance. (B) The cardiac pulse is apparent in measurement time traces. (C) A cardiac pulse peak is visible in the Fourier spectrum. (D) The pulse SNR (mean band-limited signal to noise ratio) is high across the cap with some areas of lower SNR in children likely attributable to cap fit imperfections and movement during the imaging session. (E) Comparison between the percentage of retained measurements when children are viewing movies (left) versus adults performing tasks (right). The percentage of retained measurements relates to data quality metrics such as the pulse SNR and motion. |
